# Excessive mechanical loading promotes osteoarthritis through the gremlin-1–NF-κB pathway

**DOI:** 10.1038/s41467-019-09491-5

**Published:** 2019-03-29

**Authors:** Song Ho Chang, Daisuke Mori, Hiroshi Kobayashi, Yoshifumi Mori, Hideki Nakamoto, Keita Okada, Yuki Taniguchi, Shurei Sugita, Fumiko Yano, Ung-il Chung, Joo-ri Kim-Kaneyama, Motoko Yanagita, Aris Economides, Ernesto Canalis, Di Chen, Sakae Tanaka, Taku Saito

**Affiliations:** 1 Sensory and Motor System Medicine, Graduate School of Medicine, The University of Tokyo, 7-3-1 Hongo, Bunkyo-ku, Tokyo 113-8655 Japan; 2 Bone and Cartilage Regenerative Medicine, Graduate School of Medicine, The University of Tokyo, 7-3-1 Hongo, Bunkyo-ku, Tokyo 113-8655 Japan; 3 Division of Oral Anatomy, Department of Human Development and Fostering, Meikai University School of Dentistry, 1-1 Keyakidai, Sakado, Saitama 350-0283 Japan; 4 Center for Disease Biology and Integrative Medicine, Graduate School of Medicine, The University of Tokyo, 7-3-1 Hongo, Bunkyo-ku, Tokyo 113-8655 Japan; 5 Department of Biochemistry, Showa University School of Medicine, 1-5-8 Hatanodai, Shinagawa-ku, Tokyo 142-8555 Japan; 6 Department of Nephrology, Graduate School of Medicine, Kyoto University, 54 Shogoin Kawahara-cho, Sakyo-ku, Kyoto 606-8507 Japan; 7 Regeneron Pharmaceuticals, Inc., 777 Old Saw Mill River Road, Tarrytown, NY 10591 USA; 8 Departments of Orthopaedic Surgery and Medicine and the Musculoskeletal Institute, UConn Heath, Farmington, CT 06030 USA; 9 Department of Orthopedic Surgery, Rush University Medical Center, Chicago, IL 60612 USA

## Abstract

Exposure of articular cartilage to excessive mechanical loading is deeply involved in the pathogenesis of osteoarthritis. Here, we identify gremlin-1 as a mechanical loading-inducible factor in chondrocytes, detected at high levels in middle and deep layers of cartilage after cyclic strain or hydrostatic pressure loading. Gremlin-1 activates nuclear factor-κB signalling, leading to subsequent induction of catabolic enzymes. In mice intra-articular administration of gremlin-1 antibody or chondrocyte-specific deletion of *Gremlin-1* decelerates osteoarthritis development, while intra-articular administration of recombinant gremlin-1 exacerbates this process. Furthermore, ras-related C3 botulinum toxin substrate 1 activation induced by mechanical loading enhances reactive oxygen species (ROS) production. Amongst ROS-activating transcription factors, RelA/p65 induces *Gremlin-1* transcription, which antagonizes induction of anabolic genes such as *Sox9*, *Col2a1*, and *Acan* by bone morphogenetic proteins. Thus, gremlin-1 plays essential roles in cartilage degeneration by excessive mechanical loading.

## Abstract (web summary)

Excessive mechanical stress promotes the development of osteoarthritis. Here Chang et al. identify gremlin-1 as a factor expressed in chondrocytes in response to mechanical stress, and contributing to osteoarthritis via activation of the NF-κB pathway.

## Introduction

Osteoarthritis (OA) is the most prevalent joint disorder occurring with articular cartilage degradation. Since the establishment of experimental mouse models with surgically induced knee joint instability, a large number of studies have revealed the major molecules or signalling pathways responsible for OA, such as a disintegrin-like and metallopeptidase with a thrombospondin type 1 motif 5 (Adamts5), matrix metalloproteinase-13 (Mmp13), hedgehog signalling, syndecan-4, Wnt signalling, and hypoxia-inducible factor 2-alpha (HIF-2α)^1–13^. In particular, Mmp13 is responsible for degradation of type 2 collagen (Col2a1), a major matrix protein component of articular cartilage, and plays essential roles in OA development^6,10^.

The nuclear factor kappa-light-chain-enhancer of activated B cells (NF-κB) protein complex plays essential roles in various biological processes including cell survival, proliferation, differentiation, apoptosis, aging, inflammation, and immune responses^14–16^. It consists of v-rel reticuloendotheliosis viral oncogene homologue A (RelA, also known as p65), RelB, Rel, p105/p50, and p100/p52. These proteins form heterodimers to function as transcriptional activators. Inhibitors of NF-κB (IκB) proteins, including IκBα, IκBβ, IκBγ, IκBε, IκBζ and Bcl-3, bind and sequester NF-κB family members within the cytoplasm^17^. In response to one of several signals, activation of IκB kinases (IKKs) results in phosphorylated IκB proteins, which causes their degradation to enable free NF-κB complexes to translocate from the cytoplasm into the nucleus where they trigger target gene transactivation^18^. NF-κB signalling, which is widely involved in OA pathophysiology through various effects, is activated in osteoarthritic chondrocytes during aging and inflammation^19^. NF-κB signalling is essential to induce various inflammation-related factors, including Mmp proteins, inducible nitric oxide synthase (iNOS), interleukin 1 beta (IL-1β), tumour necrosis factor alpha (TNF-α), and HIF-2α^8,9^. HIF-2α further induces various catabolic enzymes and OA-related genes^8,9^. Recently, we showed that NF-κB signalling regulates articular cartilage homoeostasis and degeneration in a biphasic manner^20^. Although NF-κB signalling is inactivated in normal articular chondrocytes, a small amount of intranuclear RelA is required for transcriptional induction of anti-apoptotic genes that are indispensable for chondrocyte survival^20^. Phosphorylated IκBα and increased intranuclear RelA accompany cartilage degeneration, leading to induction of catabolic and inflammatory molecules and acceleration of OA development^20^.

In addition to molecular biology research, clinical and epidemiologic studies previously revealed various factors to be associated with OA pathogenesis, including aging, obesity, joint instability, trauma, and joint inflammation. Excessive mechanical loading is regarded as the essence of several of these factors. A previous in vitro experiment using cell-stretcher systems showed induction of *MMP13* by excessive mechanical loading^21^. NF-κB signalling is also regulated by mechanical loading^19,22^. However, molecular mechanisms underlying cartilage degeneration by excessive mechanical loading remain unknown.

Here, we describe a signalling pathway linking excessive mechanical loading to cartilage degeneration. We perform a screen for genes altered by mechanical loading, and focus on the NF-κB-related gene *Gremlin-1* among the mechanical stress-inducible candidates. We examine its expression in articular cartilage, roles in in vitro and in vivo OA development, and further upstream and downstream pathways connecting excessive mechanical loading to cartilage degeneration.

## Results

### Excessive stress loading induces gremlin-1 in chondrocytes

**Fig. 1:**
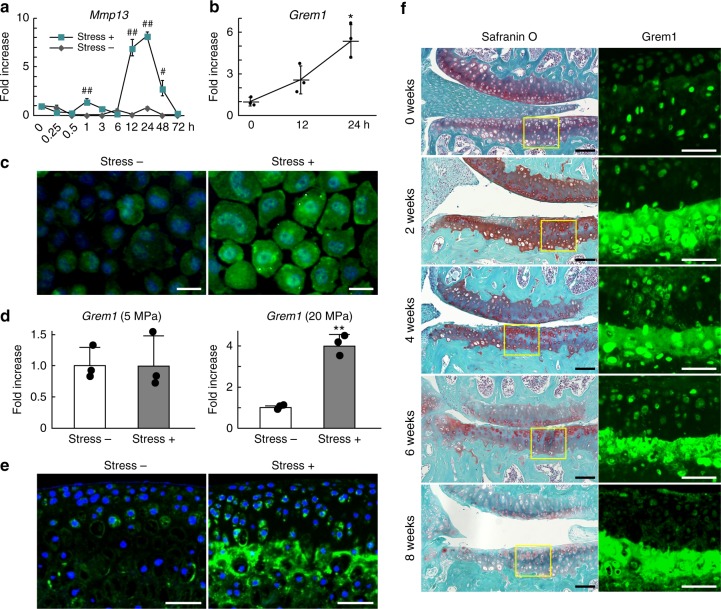
Induction of gremlin-1 by excessive mechanical loading. **a** Time-course of *Mmp13* mRNA levels in mouse primary chondrocytes after tensile stress loading (stress+), or without loading (stress −). Cells were cultured up to 72 h after uniaxial cyclic tensile strain (10%, 0.5 Hz, 30 min). ^#^*P* *<* 0.005, ^##^*P* *<* 0.001 versus stress—at each time-point (two-way ANOVA test). **b** *Gremlin-1* mRNA levels in mouse primary chondrocytes after tensile stress loading. *P < 0.05 versus 0 h (one-way ANOVA test). **c** Gremlin-1 protein expression in mouse primary chondrocytes 24 h after tensile stress loading (stress+), or without loading (stress −). Nuclei were stained with DAPI (blue). Scale bars, 50 µm. **d** *Gremlin-1* mRNA levels in mouse femoral head cartilage after hydrostatic pressure loading (5 or 20 MPa, 0.1 Hz, 30 min). ^#^*P* *<* 0.005 versus stress− (Student’s unpaired two-tailed *t*-test). **e** Gremlin-1 immunofluorescence in mouse femoral head cartilage after 20 MPa hydrostatic pressure loading. Nuclei were stained with DAPI (blue). Scale bars, 100 µm. **f** Safranin O staining and gremlin-1 immunofluorescence during the time-course of mouse osteoarthritis development after surgical induction. Inset boxes in safranin O staining indicate regions of immunofluorescence. Scale bars, 100 µm and 50 µm, respectively. All data are expressed as mean ± SD of biologically independent three samples per group

We first examined a time-course of *Mmp13* mRNA levels in mouse primary chondrocytes after 0.5 Hz, 10% cyclic tensile strain loading for 30 min. *Mmp13* mRNA expression was slightly increased 1 h after loading, peaked by 12–24 h, and declined thereafter to baseline by 72 h after loading (Fig. 1a). To identify target genes mediating *Mmp13* induction by stress loading, we performed microarray analysis using mRNA samples of chondrocytes before and 24 h after loading. Abundantly expressed genes upregulated or downregulated more than two-fold are shown in Supplementary Tables 1 and 2. Among upregulated and downregulated NF-κB-related genes identified by gene ontology analyses (Supplementary Tables 3 and 4), we focused on *gremlin-1* (*Grem1*), which showed abundant expression and was most highly upregulated by mechanical loading in the group (Supplementary Table 3). Gremlin-1 is a secreted protein and known bone morphogenetic protein (BMP) antagonist^13^. mRNA and protein levels of gremlin-1, as determined by real-time RT-qPCR and immunocytochemistry, increased after loading (Fig. 1b, c). Next, we examined *Gremlin-1* mRNA expression in mouse femoral heads under cyclic hydrostatic pressure loading by the originally developed system (Supplementary Fig. 1a–e). Although *Gremlin-1* mRNA levels were unchanged by 5 MPa loading, they were significantly increased by 20 MPa (Fig. 1d). Immunofluorescence determined that gremlin-1 protein was highly increased within the deep zone of the femoral head after 20 MPa loading (Fig. 1e). We next examined localization of gremlin-1 protein in the articular cartilage of a knee OA mouse model, which involves surgical resection of the medial meniscus and medial collateral ligament^23^. In normal mouse articular cartilage, gremlin-1 protein was predominantly localized within the deep layer (Fig. 1f). Two weeks after surgical induction, gremlin-1 was highly detected in and around deep layer chondrocytes (Fig. 1f). Its expression was slightly increased in the superficial zone after 4 weeks (Fig. 1f). We confirmed increased gremlin-1 protein levels in mouse primary chondrocytes, femoral heads, and articular cartilage using immunohistochemistry with a second primary antibody, and observed decreased protein levels using siRNA against gremlin-1 as a negative control (Supplementary Fig. 2a–d). We further examined gremlin-1 expression in human articular cartilage surgical specimens. Similar to the expression pattern in mouse cartilage, gremlin-1 protein was localized within the middle and deep layers of mildly degenerated human cartilage in the lateral tibial compartment (Supplementary Fig. 3). Gremlin-1 was intensively detected in the clustered chondrocytes in the superficial zone in severe human OA cartilage of the medial tibial compartment, as well as in and around deep layer chondrocytes (Supplementary Fig. 3).

### Gremlin-1 exerts catabolic effects in vitro

**Fig. 2:**
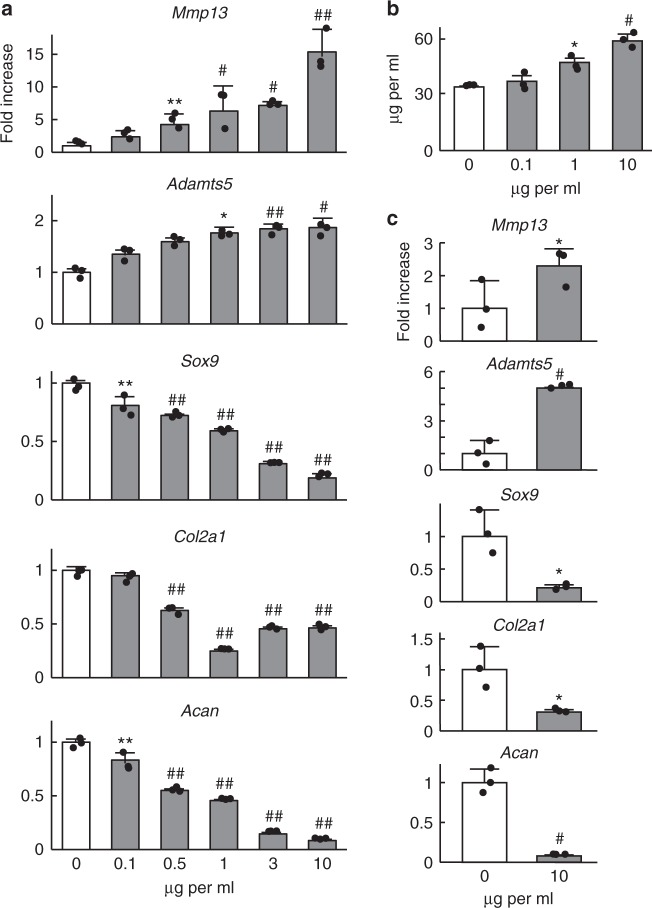
Catabolic effects of gremlin-1 in cultured chondrocytes. **a** mRNA levels of marker genes in primary mouse articular chondrocytes treated with recombinant human gremlin-1 (rhGREM1) for 24 h. **b** Amount of glycosaminoglycans (GAG) released into the culture medium determined by dimethylmethylene blue assay of wild-type mouse femoral heads cultured with various amounts of rhGREM1 for 3 days. **c** mRNA levels of marker genes in mouse femoral heads cultured with 10 µg/mL rhGREM1 for 3 days. All data are expressed as mean ± SD of biologically independent three samples per group. *P < 0.05, **P < 0.01, ^#^*P* < 0.005, ^##^*P* < 0.001 versus 0 (vehicle) (one-way ANOVA test)

To investigate the effects of gremlin-1, which is increased by excessive mechanical loading and/or during OA development, we administered recombinant human gremlin-1 (rhGREM1) protein to cultured chondrocytes. In mouse primary chondrocytes, catabolic marker genes such as *Mmp13* and *Adamts5* were increased, while *Sox9* (a master transcription factor for chondrocytes) and cartilage matrix genes *Col2a1* and *Acan* were decreased by rhGREM1 in a dose-dependent manner, and time-dependent manner up to 24 h (Fig. 2a, and Supplementary Fig. 4). Similar results were obtained using rhGREM1 manufactured by a second company (Supplementary Fig. 5). Type 10 collagen (*Col10a1*), a hypertrophic chondrocyte marker, was not changed by rhGREM1 (Supplementary Fig. 6). In ex vivo cultures of femoral head cartilage from wild-type (WT) mice, release of proteoglycans into the medium was increased by rhGREM1 treatment in a dose-dependent manner (Fig. 2b), while mRNA levels of marker genes within femoral heads were altered similarly to cultured chondrocytes (Fig. 2c).

### Gremlin-1 exerts catabolic effects on mouse cartilage

**Fig. 3:**
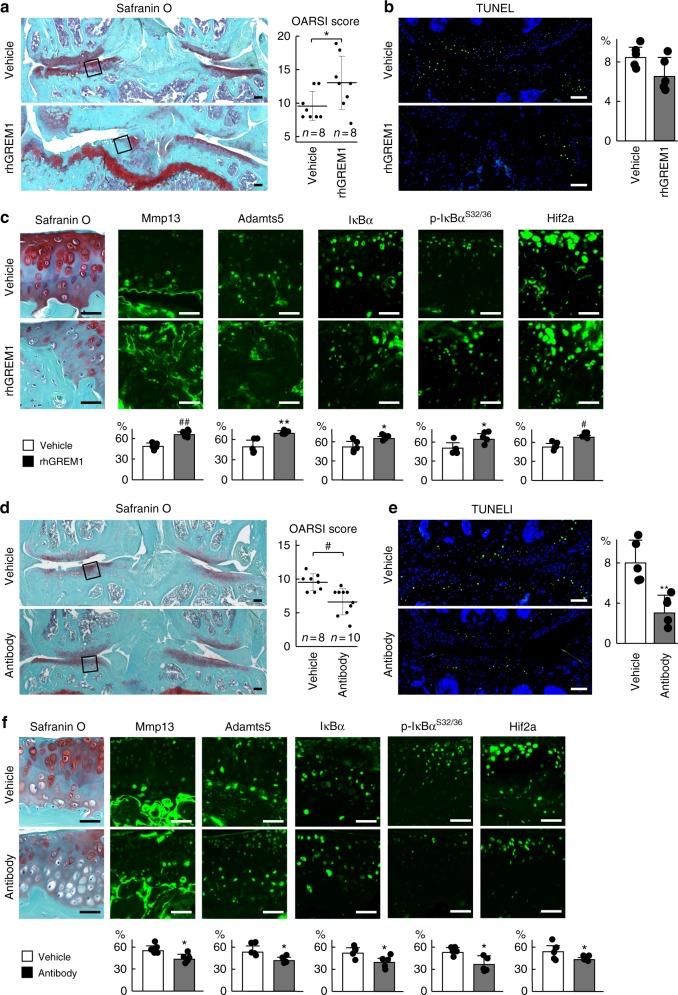
Effects of recombinant human gremlin-1 (rhGREM1) and gremlin-1 antibody in vivo. **a** Safranin O staining and OARSI scores of mouse knee joints after intra-articular administration (twice a week for 8 weeks) of 10 µL of 10 µg/mL rhGREM1 or vehicle. Both experimental groups consist of *n* = 8 biologically independent animals. Inset boxes indicate regions of immunofluorescence in (**c**). Scale bars, 100 µm. **b** TUNEL staining and rate of TUNEL-positive cells in mouse knee joints after intra-articular administration of rhGREM1 or vehicle. Nuclei were stained with DAPI (blue). Scale bars, 100 µm. *n* = 5 biologically independent experiments. **c** Safranin O staining and immunofluorescence of Mmp13, Adamts5, IκBα, phosphorylated IκBα (dual Ser32/36), and HIF-2α proteins in mouse knee joints after intra-articular administration of rhGREM1 or vehicle. Scale bars, 50 µm. The percentage of positive cells in the immunofluorescence is shown below. *n* = 5 biologically independent experiments. **d** Safranin O staining and OARSI scores of mouse knee joints after with intra-articular administration (twice a week for 8 weeks) of 10 µL of 10 µg/mL gremlin-1 antibody or vehicle. Both experimental groups consist of biologically independent animals: vehicle *n* = 8, antibody *n* = 10. Inset boxes indicate regions of immunofluorescence in (**f**). Scale bars, 100 µm. **e** TUNEL staining and rate of TUNEL-positive cells in mouse knee joints after intra-articular administration of gremlin-1 antibody or vehicle. Nuclei were stained with DAPI (blue). Scale bars, 100 µm. *n* = 5 biologically independent experiments. **f** Safranin O staining and immunofluorescence of Mmp13, Adamts5, IκBα, phosphorylated IκBα (dual Ser32/36), and HIF-2α proteins in mouse knee joints after intra-articular administration of gremlin-1 antibody or vehicle. Scale bars, 50 µm. The percentage of positive cells in the immunofluorescence is shown below. *n* = 5 biologically independent experiments. All data are expressed as mean ± SD. *P < 0.05, **P < 0.01, ^#^*P* < 0.005, ^##^*P* < 0.001 versus vehicle (Student’s unpaired two-tailed *t*-test)

We next examined the effects of gremlin-1 in vivo. The surgical OA model was prepared by injecting rhGREM1 into the knee joints of WT C57BL/6J male mice at 8 weeks of age, twice a week. Eight weeks after surgical induction, OA development was significantly enhanced by intra-articular administration of rhGREM1 (Fig. 3a). Chondrocyte apoptosis determined by TUNEL staining was unchanged by rhGREM1 treatment (Fig. 3b). Mmp13, Adamts5, IκBα, phosphorylated IκBα, and HIF-2α were highly detected in the remnant cartilage of rhGREM1-treated groups (Fig. 3c). Moreover, intra-articular injection of rhGREM1-induced OA development even in normal mouse knee joints (Supplementary Fig. 7).

In a contrasting approach, we used an antibody against gremlin-1. To confirm the neutralizing effect of the commercially obtained antibody, we treated mouse primary chondrocytes with rhGREM1 and gremlin-1 antibody. rhGREM1-induced increases in *Mmp13* and *Adamts5* were significantly reduced by antibody treatment (Supplementary Fig. 8). We then injected the antibody into the knee joints of OA model mice twice a week. Eight weeks after surgery, OA progression was significantly suppressed by antibody injection (Fig. 3d). Moreover, chondrocyte apoptosis, expression of catabolic marker proteins, and phosphorylated IκBα were also decreased (Fig. 3e, f).

### Conditional knockout of *gremlin-1* suppresses OA development

**Fig. 4:**
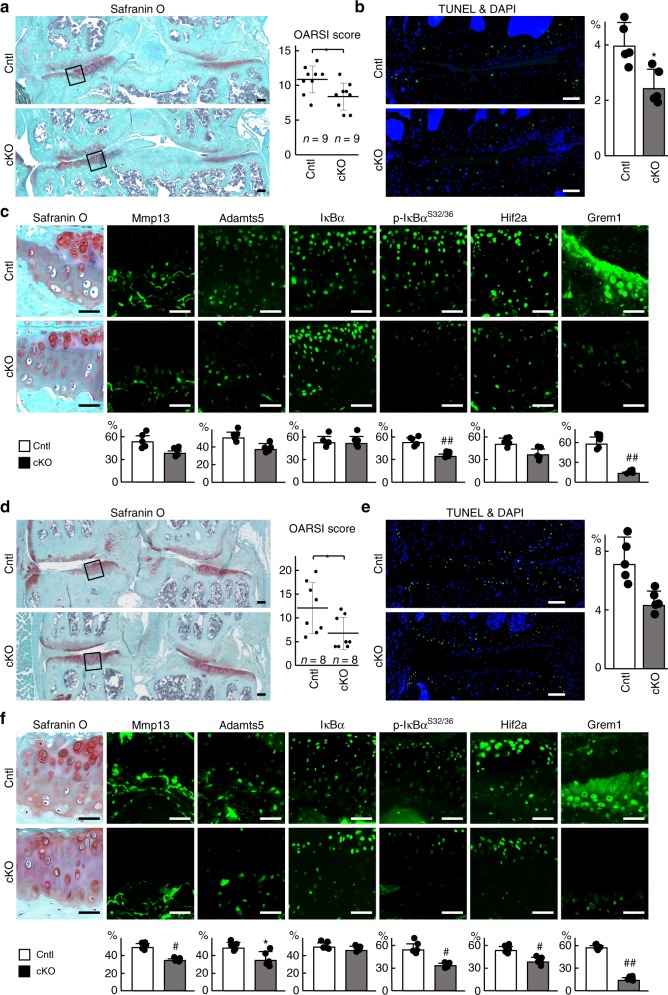
Regulation of osteoarthritis development by gremlin-1. **a** Safranin O staining and OARSI scores of mouse knee joints of *Grem1*^*fl/fl*^ (Cntl) and *Col2a1-Cre*^*ERT2*^*;Grem1*^*fl/fl*^ (cKO) mice 8 weeks after surgery. Tamoxifen induction was performed at 7 weeks. Both experimental groups consist of *n* = 9 biologically independent animals. Inset boxes indicate regions of immunofluorescence in (**c**). Scale bars, 100 µm. **b** TUNEL staining and rate of TUNEL-positive cells in mouse knee joints of Cntl and cKO mice 8 weeks after surgery. Nuclei were stained with DAPI (blue). Scale bars, 100 µm. *n* = 5 biologically independent experiments. **c** Safranin O staining and immunofluorescence of Mmp13, Adamts5, IκBα, phosphorylated IκBα (dual Ser32/36), HIF-2α, and gremlin-1 proteins in mouse knee joints of Cntl and cKO mice 8 weeks after surgery. Scale bars, 50 µm. The percentage of positive cells in the immunofluorescence is shown below. *n* = 5 biologically independent experiments. **d** Safranin O staining and OARSI scores of mouse knee joints of Cntl and cKO mice at 18 months of age. Tamoxifen induction was performed for 5 days at 8 weeks, 6 months, and 12 months. Both experimental groups consist of *n* = 8 biologically independent animals. Inset boxes indicate regions of immunofluorescence in (**f**). Scale bars, 100 µm. **e** TUNEL staining and rate of TUNEL-positive cells in mouse knee joints of Cntl and cKO mice at 18 months of age. Nuclei were stained with DAPI (blue). Scale bars, 100 µm. *n* = 5 biologically independent experiments. (**f**) Safranin O staining and immunofluorescence of Mmp13, Adamts5, IκBα, phosphorylated IκBα (dual Ser32/36), HIF-2α, and gremlin-1 proteins in mouse knee joints of Cntl and cKO mice at 18 months of age. Scale bars, 50 µm. The percentage of positive cells in the immunofluorescence is shown below. *n* = 5 biologically independent experiments. All data are expressed as mean ± SD. *P < 0.05, **P < 0.01, ^#^*P* < 0.005, ^##^*P* < 0.001 versus Cntl (Student’s unpaired two-tailed *t*-test)

To reveal the physiological roles of gremlin-1 in articular cartilage, we performed in vivo loss-of-function analyses using transgenic mice in which Cre recombinase was fused to a mutated ligand-binding domain of the human oestrogen receptor driven by the *Col2a1* promoter (*Col2a1-Cre*^*ERT2*^) to knockout gremlin-1 in adult articular cartilage after skeletal growth. Thus, administration of the oestrogen antagonist tamoxifen resulted in translocation of the fusion protein into nuclei causing gene targeting. We generated tamoxifen-inducible chondrocyte-specific homozygous gremlin-1 knockout mice by mating *Col2a1-Cre*^*ERT2*^ mice with *Grem1*^*fl/fl*^ mice (*Col2a1-Cre*^*ERT2*^*;Grem1*^*fl/fl*^). *Col2a1-Cre*^*ERT2*^*;Grem1*^*fl/fl*^ (cKO) mice developed normally and displayed no skeletal abnormalities compared with *Grem1*^*fl/fl*^ (Cntl) littermates (Supplementary Fig. 9). Tamoxifen was injected into 7-week-old cKO and Cntl littermates daily for 5 days, and the surgical OA model was induced 2 days after the last injection. Eight weeks after surgical induction, OA development was significantly inhibited in cKO knee joints compared with Cntl joints (Fig. 4a). Chondrocyte apoptosis and expression of catabolic enzymes HIF-2α and phosphorylated IκBα were suppressed by gremlin-1 knockout (Fig. 4b, c).

We further examined OA development with aging. Tamoxifen was injected into cKO and Cntl littermates daily for 5 days at 8 weeks, 6 months, and 12 months. At 18 months of age, cKO mice were significantly resistant to cartilage degeneration compared with control littermates (Fig. 4d), and showed no skeletal abnormalities (Supplementary Fig. 9). Chondrocyte apoptosis and expression of catabolic enzymes HIF-2α and phosphorylated IκBα were suppressed by gremlin-1 knockout, similar to results observed in the surgical OA model (Fig. 4e, f). Efficient knockout of gremlin-1 in articular chondrocytes was confirmed at both mRNA and protein levels (Fig. 4c, f, Supplementary Figs. 10, and 11).

### Gremlin-1 activates NF-κB signaling

**Fig. 5:**
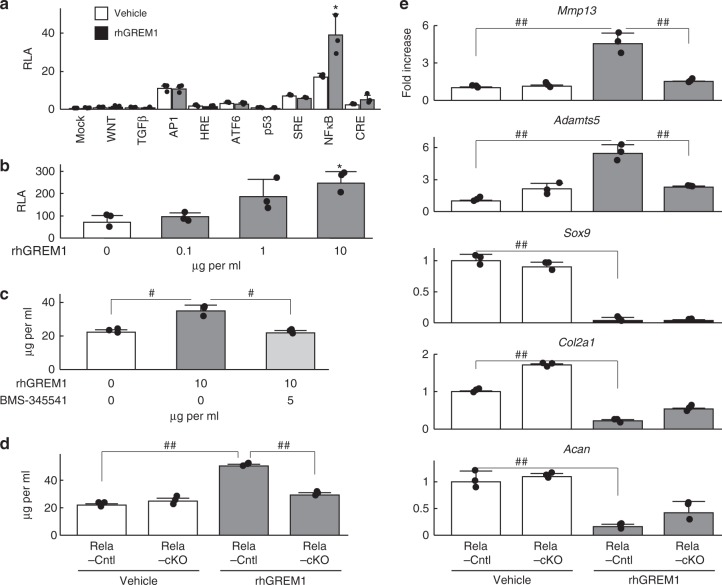
NF-κB signalling mediates the catabolic effects of gremlin-1. **a** Luciferase assay for screening of downstream signalling pathways of gremlin-1 using reporter vectors containing a response element for each pathway or transcription factor. *P < 0.05 versus each vehicle control (one-way ANOVA test). **b** Activities of luciferase reporter vectors containing an NF-κB motif with rhGREM1 treatment. *P < 0.05 versus 0 (vehicle) (one-way ANOVA test). **c** Amount of proteoglycan released into culture medium from wild-type mouse femoral heads cultured with or without rhGREM1 (10 µg/mL) and IKK inhibitor BMS-345541 (5 µM) for 3 days. ^#^*P* < 0.005 (one-way ANOVA test). **d** Amount of proteoglycan released into culture medium from *RelA*^*fl/fl*^ (Rela-Cntl) and *Col2a1-Cre;RelA*^*fl/fl*^ (Rela-cKO) mice femoral heads cultured with or without 10 µg/mL rhGREM1. ^##^*P* < 0.001 (one-way ANOVA test). **e** mRNA levels of marker genes in RelA-Cntl and RelA-cKO femoral heads cultured with or without rhGREM1. ^##^*P* < 0.001 (one-way ANOVA test). All data are expressed as mean ± SD of biologically independent three samples per group

We further analysed molecular mechanisms underlying the catabolic effects of gremlin-1 in articular cartilage by performing a luciferase assay using reporter vectors containing response elements for representative signalling pathways. Among them, the NF-κB reporter vector showed the highest transactivity induced by rhGREM1 (Fig. 5a). When various amounts of rhGREM1 were applied to cells transfected with the NF-κB reporter vector, transactivity was increased in a dose-dependent manner (Fig. 5b). In addition to rhGREM1, we next treated mouse femoral head cartilage with the IKK inhibitor BMS-345541. Increased release of proteoglycans into the medium by rhGREM1 treatment was significantly inhibited by BMS-345541 to a level similar to control (Fig. 5c). Femoral heads from *RelA*^*fl/fl*^ (RelA-Cntl) and *Col2a1-Cre;RelA*^*fl/fl*^ (RelA-cKO) mice were also treated with rhGREM1. Release of proteoglycans was enhanced by rhGREM1 in RelA-Cntl femoral heads; however, it was not enhanced in RelA-cKO femoral heads (Fig. 5d). Moreover, rhGREM1-induced increases in *Mmp13* and *Adamts5* were not observed in RelA-cKO femoral heads, and decreases in anabolic marker genes including *Sox9*, *Col2a1*, and *Acan* were not rescued (Fig. 5e). These data indicate that gremlin-1 induces catabolic enzymes through NF-κB signalling, but does not suppress anabolic genes in the same pathway.

We next examined which molecule functions as a receptor for gremlin-1 to stimulate NF-κB signalling. Among several candidates from previous reports, we focused on vascular endothelial growth factor receptor 2 (VEGFR2), which is known to bind to gremlin-1^24,25^. Using the selective VEGFR2 inhibitor SU5416 in combination with rhGREM1, we found that increased *Mmp13* and *Adamts5* by rhGREM1 were suppressed by SU5416 in a dose-dependent manner (Supplementary Fig. 12a). Moreover, SU5416 did not affect expression of *Sox9*, *Col2a1*, or *Acan*, similar to RelA-cKO (Fig. 5e, Supplementary Fig. 12a). The rate of p-VEGFR2-positive cells increased 10 min after rhGREM1 treatment, while that of VEGFR2-positive cells was unaltered (Supplementary Fig. 12b). mRNA levels of VEGF family members were not upregulated by rhGREM1 treatment in mouse primary chondrocytes (Supplementary Fig. 13).

### Gremlin-1 antagonizes the anabolic effects of BMPs

**Fig. 6:**
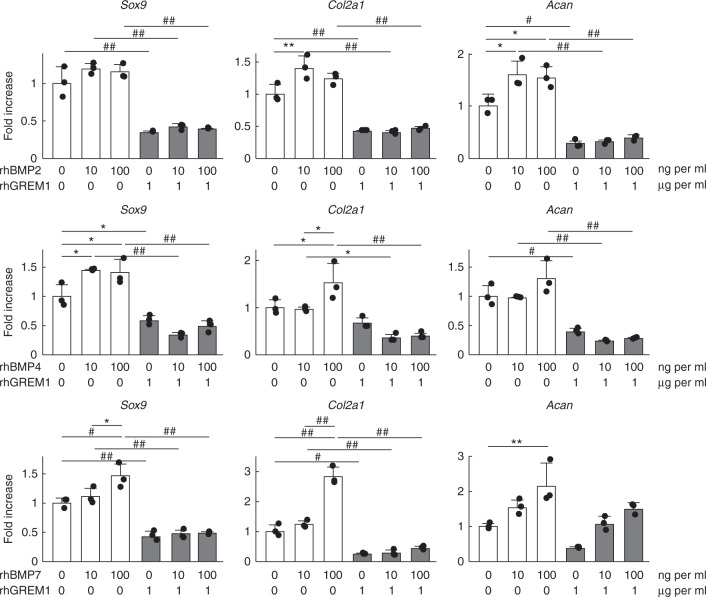
Gremlin-1 antagonizes the anabolic effects of BMPs. mRNA levels of *Sox9*, *Col2a1* and *Acan* in mouse primary chondrocytes treated with rhBMP2, 4, or 7, and rhGREM1 for 24 h. *P < 0.05, **P < 0.01, ^#^*P* < 0.005, ^# #^*P* < 0.001 versus vehicle (one-way ANOVA test). All data are expressed as means ± SD of biologically independent three samples per group

Gremlin-1 treatment increased the expression of catabolic enzymes *Mmp13* and *Adamts5*, while decreasing the expression of cartilage matrix genes *Col2a1* and *Acan* (Fig. 2a). Among these alterations, inhibition of NF-κB signalling almost completely suppressed the increase of catabolic enzymes by gremlin-1 (Fig. 5c–e), but it did not affect observed decreases of anabolic factors (Fig. 5e). We next examined antagonistic effects between gremlin-1 and BMPs. In mouse primary chondrocytes, rhBMP-2, rhBMP-4, and rhBMP-7 enhanced the expression of anabolic markers (Fig. 6). Anabolic effects elicited by rhBMPs were diminished by rhGREM1 treatment (Fig. 6).

### Gremlin-1 is induced by mechanical loading through Rac1

**Fig. 7:**
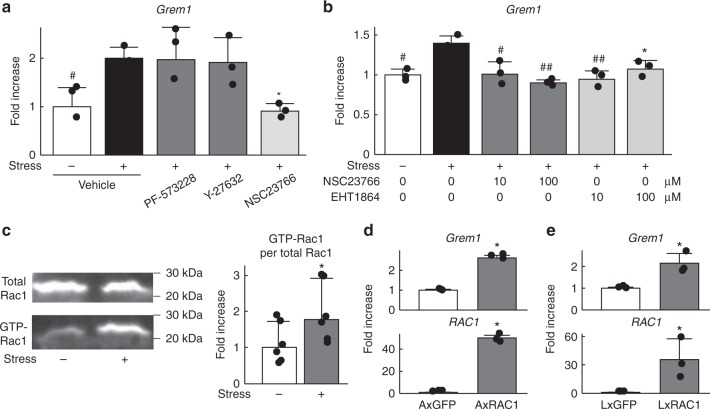
Gremlin-1 induction by mechanical stress loading occurs through Rac1 activation. **a** *Gremlin-1* mRNA levels in mouse primary chondrocytes treated with 10 µM inhibitors of FAK (PF-573228), ROCK (Y-27632), or RAC1 (NSC23766) 24 h after cyclic tensile strain loading. *n* = 3 biologically independent samples. *P < 0.05, ^#^*P* < 0.005 versus stress+, vehicle (one-way ANOVA test). **b** *Gremlin-1* mRNA levels in mouse primary chondrocytes treated with Rac1 inhibitors NSC23766 or EHT1864 at 24 h after cyclic tensile strain loading. *n* = 3 biologically independent samples. *P < 0.05, ^#^*P* < 0.005, ^##^*P* < 0.001 versus stress + , vehicle (one-way ANOVA test). **c** Rac1 pull-down activation assay using mouse primary chondrocytes with or without cyclic tensile strain loading. Quantification of densitometry data are shown below, and ratios of positive Rac1 per total Rac1 are shown as fold-increase in the right graph. *n* = 5 biologically independent samples. *P < 0.05 versus stress– (Student’s unpaired two-tailed *t*-test). **d** *Gremlin-1* mRNA levels in mouse primary chondrocytes transduced with an adenoviral vector containing *Rac1* or *GFP*. *n* = 3 biologically independent samples. *P < 0.05 versus *GFP* (Student’s unpaired two-tailed *t*-test). **e** *Gremlin-1* mRNA levels in ATDC5 cells lentivirally overexpressing *Rac1* or *GFP*. *n* = 3 biologically independent samples. *P < 0.05 versus *GFP* (Student’s unpaired two-tailed *t*-test). All data are expressed as means ± SD

Finally, we searched for an upstream pathway of gremlin-1 induction by mechanical loading using a screen of selective inhibitors of mechanotransduction-related molecules, such as focal adhesion kinase (FAK), Rho-associated coiled-coil forming kinase (ROCK), and ras-related C3 botulinum toxin substrate 1 (Rac1). NSC23766, a specific inhibitor of the binding and activation of Rac guanosine triphosphate-binding protein (GTPase), decreased gremlin-1 induction by cyclic tensile strain loading, although a FAK inhibitor (PF-573228) and ROCK inhibitor (Y-27632) had no effect (Fig. 7a). EHT1864, another inhibitor of Rac family GTPases, also significantly decreased gremlin-1 induction by loading, similar to NSC23766 (Fig. 7b). An active Rac1 pull-down assay confirmed Rac1 activation by mechanical loading (Fig. 7c). Moreover, gremlin-1 expression was significantly increased by adenoviral or lentiviral overexpression of Rac1 in mouse primary chondrocytes or ATDC5 cells, respectively (Fig. 7d, e).

**Fig. 8:**
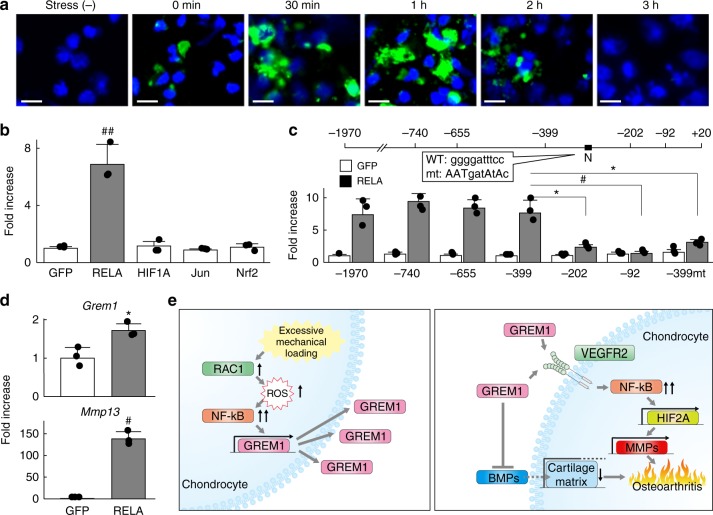
The ROS- NF-κB pathway enhances transcriptional induction of gremlin-1. **a** Fluorescence imaging time-course of ROS production in mouse primary chondrocytes after cyclic tensile strain loading. Nuclei were stained with DAPI (blue). Scale bars, 20 µm. **b** Luciferase assay using ATDC5 cells co-transfected with human *GREM1* promoter (from −1970 to +20 bp relative to the transcriptional start site) and each expression vector. ^##^*P* < 0.001 versus *GFP* (one-way ANOVA test). **c** 5ʹ-deletion and mutation analyses of the luciferase assay. N: NF-κB motif, −399mt: reporter construct from −399 to +20 bp in which NF-κB motif is mutated. *P < 0.05, ^#^*P* < 0.005 (Student’s unpaired two-tailed *t*-test). **d** mRNA levels of *Gremlin-1* and *Mmp13* in mouse primary chondrocytes transfected with *RelA* or *GFP*. *P < 0.05, ^#^*P* < 0.005 versus *GFP* (Student’s unpaired two-tailed *t*-test). **e** Schematic diagram representing molecular pathways in which excessive mechanical loading induces osteoarthritis development through gremlin-1. All data are expressed as mean ± SD of biologically independent three samples per group

Rac1 is widely known to act upstream of reactive oxygen species (ROS) production in a variety of cell types^26^. Thus, we next examined in situ ROS production in mouse primary articular chondrocytes after cyclic tensile strain loading. ROS was detected immediately after loading for 30 min, increased until 1 h, and was diminished at 3 h (Fig. 8a). Several transcription factors including NF-κB, HIF-1α, c-Jun, and nuclear factor erythroid 2-related factor 2 (Nrf2) are activated by the Rac1-ROS axis^27^. As such, we prepared a luciferase reporter vector containing a 5ʹ-end flanking region (from −1970 to +20 bp relative to the transcriptional start site) of human *GREM1*, and performed a luciferase assay by co-transfection with expression vectors for *RelA*, *HIF-1α*, *c-Jun*, or *Nrf2*. Among the four transcription factors, only RelA markedly enhanced activity of the *GREM1* proximal promoter (Fig. 8b). This region contains one NF-κB motif (−279 to −270 bp) and three sequences similar to the NF-κB motif (Fig. 8c). Deletion analysis by a series of 5ʹ-deletion constructs identified the core responsive element to RelA to be between −399 and −202 bp (Fig. 8c). Site-directed mutagenesis of the NF-κB motif in the reporter construct containing the region between −399 to +20 bp inhibited the enhanced activity by RelA, indicating it is the core responsive element of RelA (Fig. 8c). Furthermore, gremlin-1 expression was significantly increased by adenoviral overexpression of RelA in mouse primary chondrocytes (Fig. 8d). Previously, we generated adult chondrocyte-specific *RelA* knockout mice^20^. In hetero-knockout and homo-knockout cartilage, gremlin-1 expression was decreased compared with respective control cartilage (Supplementary Fig. 14). These data indicate that excessive mechanical loading enhances *Gremlin-1* transcription through the Rac1–ROS–NF-κB pathway (Fig. 8e).

## Discussion

The present study identified gremlin-1 as a key factor mediating cartilage degeneration by excessive mechanical loading. Gremlin-1 activates NF-κB signalling, which results in induction of catabolic enzymes such as Mmp13 and Adamts5. The Rac1–ROS–NF-κB axis plays an essential role in gremlin-1 induction by excessive mechanical loading. Genetic deletion or neutralization of gremlin-1 by intra-articular injection of an antibody suppressed OA development in mice.

Gremlin-1 is a well-known secreted BMP antagonist that was formerly identified as a regulator of limb formation^11–13^. Gremlin-1 exerts potent inhibitory action via binding to and forming heterodimers with BMP-2, BMP-4, and BMP-7^13^. Binding to selective BMPs prevents ligand–receptor interactions and subsequent downstream signalling^13^. Indeed, gremlin-1 regulates development of limb, lung, kidney and retina through BMP inhibition^28–30^. In addition to its inhibitory effect on BMP, gremlin-1 exerts direct effects on cell function via BMP-independent mechanisms. For example, VEGFR2 was identified as a novel receptor for gremlin-1^24^. The gremlin-1–VEGFR2 axis exerts a proinflammatory effect^25^, which is mediated by activation of NF-κB signalling^31^. In addition to its roles in the development of various organs, gremlin-1 has been implicated in many diseases and pathogenic mechanisms, such as heart, lung and liver fibrosis, as well as osteogenesis, angiogenesis and cancer—some of which may be mediated by this axis^32^. Considering that the induction of catabolic factors by gremlin-1 was diminished by inhibition of VEGFR2 or NF-κB in the present data, the axis is also responsible for cartilage degeneration by gremlin-1. However, we cannot exclude the possibility that carrier proteins in rhGREM1 solution may exert some catabolic effects in intra-articular injection experiments.

In contrast to these catabolic effects, downregulation of cartilage matrix gene expression by gremlin-1 was not dependent on the NF-κB pathway. Moreover, the present in vitro data revealed that gremlin-1 suppressed induction of *Sox9*, *Col2a1*, and *Acan* by rhBMPs (Fig. 6). BMPs play essential roles in bone and cartilage formation, and have been extensively used in cartilage regenerative research^33^. Exogenous BMP-2 or BMP-4 enhance chondrocyte differentiation of stem cells and cartilage matrix production by chondrocytes^34–36^. BMP-7 also exhibits chondrogenic effects and the ability to repair cartilage and suppress cartilage degeneration^37,38^. Meanwhile, gremlin-1 consistently demonstrated a higher binding affinity for BMP-2 > BMP-4 > BMP-7^39^. Although we could not determine specific targets of gremlin-1 among BMP family members, gremlin-1 may affect articular cartilage homeostasis through downregulation of anabolic genes by antagonizing BMPs.

BMPs are also involved in hypertrophic differentiation during endochondral ossification. BMP-2, which is highly expressed in the hypertrophic zone, promotes hypertrophic differentiation of chondrocytes in the proliferative zone^40^. BMP-4 expression is very low in the growth plate, while BMP-7 is more highly expressed in the proliferative zone than in the hypertrophic zone^40^. Hypertrophic differentiation is involved in OA development;^41^ however, it is not well understood how each endogenous BMP produced from articular chondrocytes or neighbouring cells regulates their hypertrophic differentiation. In the present study, Col10a1 expression was decreased by cyclic tensile strain loading (Supplementary Table 2), but not altered by rhGREM1 treatment in mouse primary chondrocytes (Supplementary Fig. 6). Although we cannot determine the effect size, hypertrophic differentiation seems to be less important in the regulation of OA development by gremlin-1, compared with the catabolic effects enacted through the gremlin-1–NF-κB axis.

The Rho family of small GTPases, such as Rac, Cdc42, and RhoG, are master regulators of many cellular activities^42,43^. Conformationally regulated by the binding of GTP and GDP, GTPases actively function with their downstream target proteins when bound to GTP^43^. Among various types of stimulants, mechanical loading activates Rac1 through effects on the actin cytoskeleton^26,44^. Rac1 binds the complex of nicotinamide adenine dinucleotide phosphate (NADPH) oxidase enzymes to enhance ROS production in a variety of cell types in response to mechanical loading^26,45^. Activation of the Rac1-ROS pathway enhances transcription of several molecules, such as NF-κB, HIF-1α, c-Jun, and Nrf2^27^. Amongst upregulated transcription factors, RelA strongly induced gremlin-1 expression (Fig. 8b). Luciferase assays revealed functional NF-κB consensus motifs within the proximal 5ʹ-flanking region of the *GREM1* gene (Fig. 8c). In turn, gremlin-1 exerts catabolic effects through the NF-κB pathway. In fact, *Mmp13* mRNA levels are slightly and transiently increased one hour after stress loading, and peak by 12–24 h after loading (Fig. 1a). Early and late increases of *Mmp13* mRNA may result from activation of NF-κB signalling by Rac1-ROS and gremlin-1, respectively (Fig. 8e). Thus, iterated intensive mechanical loading causes gremlin-1 protein to excessively accumulate within the deep layer of articular cartilage, where it may function to amplify mechanical stress-induced NF-κB activation. However, it is difficult to discern the entire hierarchical relationship between these molecules in one experimental system. The hypothesis shown in Fig. 8e should be examined by following studies in the future.

In conclusion, we found that induction of gremlin-1 by excessive mechanical loading occurs via the Rac1-ROS-RelA/p65 axis, which accelerates OA development mainly through the NF-κB pathway. Our findings contribute to understanding of the pathophysiology of various diseases in which mechanical loading, gremlin-1, and NF-κB are involved, including OA.

## Methods

### Cell cultures

Mouse chondrogenic ATDC5 cells (RIKEN Cell Bank) were grown and maintained in Dulbecco’s Modified Eagle’s Media with F-12 supplement (DMEM/F-12, 1:1) with 5% fetal bovine serum (FBS). Primary articular chondrocytes were isolated from 6-day-old C57BL/6J mice according to a standard protocol using collagenase D^46^. Primary chondrocytes were cultured in DMEM with 10% FBS. The medium was changed every 3 days. For most experiments, primary cells were transferred to serum-free DMEM for 24 h before being exposed to stimuli. In some experiments, cells were pretreated with recombinant human gremlin-1 (rhGREM1, #120-42, PeproTech for Figs. 2, 3, 5, 6, Supplementary Figs. 4, 6–8, 12, and 13; 5190-GR, R&D Systems for Supplementary Fig. 5), recombinant human BMP-2 (rhBMP2, #120-02, PeproTech), recombinant human BMP-4 (rhBMP4, #120-05, PeproTech), recombinant human BMP-7 (rhBMP7, #120-03, PeproTech), IKK inhibitor BMS-345541 (B9935, Sigma), selective VEGFR2 inhibitor SU5416 (ab145056, Abcam), FAK inhibitor PF-573228 (S2013, Selleck Chemicals), ROCK inhibitor Y-27632 (257-00511, WAKO), Rac1 inhibitor NSC23766 (SML0952, Sigma) and/or EHT1864 (S7482, Selleck Chemicals). For gene silencing experiments, siRNA against mouse gremlin-1 (#1320001, Thermo-Fisher Scientific) or negative control (#12935300, Thermo Fisher Scientific) were transfected into mouse primary chondrocytes using Lipofectamine 2000 reagent (Thermo Fisher Scientific) according to the manufacturer’s protocol. Cells were harvested 24 h after transfection.

### Animals

All animal experiments were authorized by the Animal Care and Use Committee of The University of Tokyo. We have complied with all relevant ethical regulations. In each experiment, we compared the genotypes of littermates maintained in a C57BL/6J background. *Col2a1-Cre*^47^ were purchased from Jackson Laboratory. *Col2a1-Cre*^*ERT*^ mice^48^ and *RelA*^*fl/fl*^ mice^49^ were generously provided by Professor Fanxin Long (Washington University, St. Louis) and Professor Roland M. Schmid (Technical University of Munich), respectively. To generate *Col2a1-Cre;RelA*^*fl/fl*^ and *Col2a1-Cre*^*ERT*^*;RelA*^*fl/fl*^ mice, *RelA*^*fl/fl*^ mice were mated with *Col2a1-Cre* or *Col2a1-Cre*^*ERT*^ mice to obtain *Col2a1-Cre;RelA*^*fl/+*^ and *Col2a1-Cre*^*ERT*^*;RelA*^*fl/+*^ mice, respectively, which were then mated with *RelA*^*fl/fl*^ mice. *Col2a1-Cre*^*ERT2*^ mice and *Grem1*^*fl/fl*^ mice were generated as previously described^50,51^. To generate *Col2a1-Cre*^*ERT2*^*;Grem1*^*fl/fl*^ mice, *Grem1*^*fl/fl*^ mice were mated with *Col2a1-Cre*^*ERT2*^ mice to obtain *Col2a1-Cre*^*ERT2*^*; Grem1*^*fl/+*^ mice, which were then mated with *Grem1*^*fl/fl*^ mice. To confirm knockout efficiency, we directly isolated mRNA from the articular cartilage of 16-week-old *Grem1*^*fl/fl*^ and *Col2a1-Cre*^*ERT2*^*;Grem1*^*fl/fl*^ animals that had received tamoxifen administration at 7 weeks of age. For animal studies, no randomization was used.

### Human samples

We obtained samples of human articular cartilage undergoing total knee arthroplasty. Written informed consent was obtained from all individuals, and approval was provided by the ethics committee of the University of Tokyo. We have complied with all relevant ethical regulations.

### Cyclic tensile strain loading of mouse primary chondrocytes

Mouse primary chondrocytes were seeded into silicon stretch chambers coated with fibronectin at a density of 1 × 10^5^ cells/chamber; each chamber had a culture surface of 2 × 2 cm. After 48 h, cyclic tensile strain (0.5 Hz, 10% elongation) was applied for 30 min using an STB-140 mechanical stretch system (STREX, Osaka, Japan) in a CO_2_ incubator^21^. Control cells were seeded onto the same chambers and cultured without cyclic tensile strain.

### Cyclic hydrostatic pressure loading of mouse femoral head explants

The cyclic hydrostatic pressure loading system was developed by STREX. Femoral heads obtained from 3-week-old mice were put into the chamber, which consisted of gas permeable membranes with a metallic frame (Supplementary Fig. 1a); each chamber was filled with DMEM/F-12 (1:1) containing 5% FBS and set into a cylindrical container (Supplementary Fig. 1a–c). Cyclic hydrostatic compression pressure was applied by the pumping machine connected to the cylindrical containers (Supplementary Fig. 1d, e). We employed 5 and 20 MPa for normal and excessive stress loading, respectively, because maximum pressure to human knee cartilage ranges from 3 to 9 MPa during a normal gait^52–54^. We applied 5 or 20 MPa hydrostatic pressure to mouse femoral heads for 30 min at a frequency of 0.1 Hz. Control femoral heads were put into in the same chambers and cultured without cyclic hydrostatic pressure.

### qRT-PCR

Total RNA was purified with an RNeasy Mini Kit (Qiagen). One microgram of total RNA was reverse transcribed using ReverTraAce qPCR RT Master Mix with gDNA Remover (Toyobo). Each PCR reaction contained 1 × THUNDERBIRD SYBR qPCR Mix (Toyobo), 0.3 mM specific primers, and 20 ng of cDNA. Copy number was normalized to rodent total RNA (Thermo-Fisher Scientific), with rodent glyceraldehyde-3-phosphate dehydrogenase used as an internal control. All reactions were run in triplicate. Primer sequences are shown in Supplementary Table 5.

### Microarray analysis

Total RNA was isolated from mouse primary chondrocytes before and 24 h after cyclic tensile strain loading using an RNeasy Mini Kit (Qiagen). Microarray experiments were performed using SurePrint G3 Mouse Gene Expression 8 × 60 K (Agilent Technologies). Arrays were then scanned with an Agilent DNA Microarray Scanner (G2565CA), and acquired array images were analysed by Agilent Feature Extraction software (version 10.10.1.1).

### Histological analyses

Tissue samples were fixed in 4% paraformaldehyde buffered with phosphate-buffered saline (PBS, pH 7.4) at 4 °C for 1 day. Specimens were decalcified with 10% EDTA (pH 7.4) at 4 °C for 2 weeks, embedded in paraffin, and 4-µm thick sagittal sections were cut from specimens. Safranin O staining was performed according to standard protocols. For immunohistochemistry, sections were incubated with antibodies against gremlin-1 (1:250; LS-C125371, LSbio for Figs. 1e, f and 4c, f, Supplementary Figs. 3 and 14: 1:250; AF956, R&D Systems for Supplementary Figs. 2c, 2d, and 11), Mmp13 (1:200; MAB13426, Chemicon), Adamts5 (1:500; H-200, Santa Cruz Biotechnology), IκBα (1:100; sc-371, Santa Cruz Biotechnology), p-IκBα^S32/36^ (1:100; sc-101713, Santa Cruz Biotechnology), and/or HIF-2α (1:200; NB100-122, Novus Biologicals). For immunofluorescence, a CSA II Biotin-Free Catalyzed Amplification System (Agilent Technologies) and Hoechst 33258 (Agilent Technologies) counterstain were used. TdT-mediated dUTP nick end labelling (TUNEL) staining was performed with an In Situ Cell Death Detection Kit (Roche) according to the manufacturer’s instructions. Histological analyses were performed at least three times using 2–3 mice per group or genotype for confirmation of results. Images were visualized under a fluorescence microscope (BZ-X710, Keyence, Osaka, Japan). Counts of immunofluorescence-positive cells were calculated in five independent squares (100 × 100 µm) of articular cartilage harvested from the mouse medial tibial plateau, using BZ analyser software (Keyence). The same parameters were used for all acquisitions, and representative pictures are shown in the figures.

### Immunocytochemistry

Cells were fixed with 4% paraformaldehyde buffered with PBS (pH 7.4). After incubation for 10 min in PBS containing 0.1% Triton X-100 (Sigma Aldrich), cells were rinsed in PBS, and treated with PBS containing 1% bovine serum albumin (BSA; Sigma-Aldrich). Samples were stained using primary antibodies against Grem1 (1:500, LS-C125371, LSBio for Fig. 1c; 1:250, AF956, R&D Systems for Supplementary Fig. 2a, b), VEGFR2 (1:500, 55B11, Cell Signaling Technology for Supplementary Fig. 12b) and p-VEGFR2 (1:500, Tyr951, #2471, Cell Signaling Technology for Supplementary Fig. 12b) for 24 h in PBS containing 1% BSA at 4 °C, followed by AlexaFluor 488-conjugated mouse and rabbit secondary antibodies (1:500, Invitrogen) for 1 h. Slides were then treated with Vectashield Mounting Medium with DAPI (H-1200, Vector Laboratories). Images were visualized under a fluorescence microscope (BZ-X710, Keyence, Osaka, Japan). Counts of immunofluorescence- positive cells were calculated in five independent squares (100 × 100 µm) using BZ analyser software (Keyence).

### OA experiment

For surgical model, tamoxifen (Sigma; 100 µg per g of body weight) was intraperitoneally injected into 7-week-old *Col2a1-Cre*^*ERT*^*;Grem1*^*fl/fl*^ and *Grem1*^*fl/fl*^ mice daily for 5 days. A surgical procedure was then performed to establish an experimental OA model in 8-week-old male mice^23^. Under general anaesthesia, resection of the medial collateral ligament and medial meniscus was performed using a surgical microscope. Mice were analysed 8 weeks after surgery. We also analysed OA development with aging using 18-month-old mice bred under physiological conditions. For the aging experiments, tamoxifen induction was performed for 5 days at 8 weeks, 6 months, and 12 months. All mice were maintained under the same conditions (three mice per cage). OA severity was quantified by the Osteoarthritis Research Society International (OARSI) system^55^, which was assessed by two observers blinded to the experimental groups.

### Intra-articular administration of rhGREM1 or gremlin-1–neutralizing antibody

We performed intra-articular administration of rhGREM1 or gremlin-1–neutralizing antibody to C57BL/6J mice twice a week for 8 weeks after OA surgery, or normal mice without surgery. For each administration, we injected 10 µL of 10 µg/mL rhGREM1 solution diluted in PBS, 10 µL of 10 µg/mL gremlin-1–neutralizing antibody (LS-C125371, LSbio) solution diluted in PBS, or 10 µL of PBS as a vehicle control.

### Proteoglycan release assay

Proteoglycan release from mouse femoral head explants was assessed according to the standard protocol^46^. Briefly, femoral heads were harvested from 3-week-old WT or *RelA*^*fl/fl*^ and *Col2a1-Cre;RelA*^*fl/fl*^ mice, and cultured for 3 days with or without rhGREM1 (10 µg/mL) in DMEM containing 1% penicillin/streptomycin. Proteoglycan content within the medium was measured as sulfated glycosaminoglycan by a colorimetric assay using dimethylmethylene blue.

### Luciferase assay

Reporter constructs containing the human *GREM1* proximal promoter region were prepared by PCR amplification of a human genomic DNA template, which were cloned into the pGL4.10[luc2] vector (Promega). Deletion and mutation constructs were created by PCR. Transfection of ATDC5 cells was performed in triplicate in 48-well plates using Lipofectamine 2000 transfection reagent (Thermo-Fisher). The reaction mixture consisted of Lipofectamine 2000 with 400 ng of plasmid DNA, 200 ng of pGL4 reporter vector, 200 ng of effector vector, and 4 ng of pRL-TK internal control vector (Promega) per well. For screening of downstream gremlin-1 signalling, we purchased pGL4.49[luc2P/TCF-LEF RE/Hygro], pGL4.48[luc2P/SBE/Hygro], pGL4.44[*luc2P*/AP1 RE/Hygro], pGL4.42 [*luc2P*/HRE/Hygro], pGL4.39[*luc2P*/ATF6 RE/Hygro], pGL4.38[*luc2P*/p53 RE/Hygro], tpGL4.33[*luc2P*/SRE/Hygro], pGL4.32[luc2P/NF-κB-RE/Hygro], and pGL4.29[*luc2P*/CRE/Hygro] vectors (Promega). Transfections were performed as described above except with PBS and rhGREM1 (1 µg/mL) in lieu of effector vectors. To evaluate the dose-response of NF-κB activity, we used 0.1, 1, and 10 µg/mL of rhGREM1. Luciferase assays were performed with a PicaGene Dual SeaPansy Luminescence Kit (Toyo Ink) using a GloMax 96 Microplate Luminometer (Promega). Data are shown as the ratio of firefly activity to Renilla activity.

### Rac1 activation assay

To evaluate Rac1 activity, a Rac1 Pull-down Activation Assay Biochem Kit (Cytoskeleton) was employed. Mouse primary chondrocytes were plated at a density of 1 × 10^5^ cells in 2 × 2 cm stretch chambers. After cyclic tensile strain (0.5 Hz, 10% elongation) for 30 min, proteins were isolated from cells and 500 µg was used in a pull-down assay with p21-activated kinase–Rac1 p21 binding domain beads. The resulting pull-down product was then immunoblotted with Rac1 antibody according to the manufacturer’s instructions. His-tagged Rac1 protein in the kit was used for control. The membranes were incubated with a horseradish peroxidase-conjugated antibody (Promega), and visualized with ECL prime (GE Healthcare) and an AE-6981 Light Capture II (ATTO, Tokyo, Japan). Control cells were seeded onto identical chambers and cultured without cyclic tensile strain. Original images of the assay were shown in Supplementary Fig. 15.

### Construction of expression vectors

Full-length human or mouse cDNA sequences of transcription factors were PCR-amplified and cloned into pCMV-HA or pShuttle vector (Clontech). We generated adenovirus vectors using an Adeno-X expression system (Clontech) and verified all vectors by DNA sequencing.

### Fluorescence detection of ROS

Intracellular ROS accumulation was evaluated by fluorescent microscopy using a Total ROS Detection Kit (ENZ-51010, Enzo Life Sciences). Mouse primary chondrocytes were plated in 2 × 2 cm stretch chambers and cyclic tensile stress (0.5 Hz, 10% elongation) was applied for 30 min. Cells were stained with 1 µM of ROS-sensitive fluorescent dye at 37 °C for 30 min, and then washed once with 1× wash buffer. After washing, cells were immediately overlaid with a coverslip and observed under a BZ-X710 fluorescence microscope (Keyence). Fluorescence signals were subsequently detected with excitation at 490 nm and emission at 525 nm for detection of ROS.

### Statistical analyses

JMP Pro 13 was used for statistical analysis. Values are expressed as mean ± standard deviation. For comparisons between two groups, Student’s unpaired two-tailed *t*-test was applied. For multiple comparisons, statistical analysis was performed by a two-way repeated measures for variance (ANOVA) test for Fig. 1a and a one-way ANOVA followed by Tukey–Kramer honest significant difference comparison. Sample sizes were chosen based on prior literature using similar methods and reporting moderate effect size. *P* values < 0.05 were considered significant.

### Reporting Summary

Further information on experimental design is available in the Nature Research Reporting Summary linked to this article.

## Supplementary information

Supplementary Information

Reporting Summary

## Data Availability

The raw microarray data are deposited in the Gene Expression Omnibus (www.ncbi.nlm.nih.gov/geo/) under accession no. GSE103159. All other data supporting the findings of this study are available within the article and the Supplementary Information file, or are available from the corresponding author on reasonable request.
