# Supplementary material for: Excessive mechanical loading promotes osteoarthritis through the gremlin-1–NF-κB pathway
Source: Nat Commun. 2019 Mar 29;10:1442. doi: 10.1038/s41467-019-09491-5 (PMC6441020; doi:10.1038/s41467-019-09491-5)
Supplement: Supplementary file 1 — Supplementary Information [file 41467_2019_9491_MOESM1_ESM.pdf]

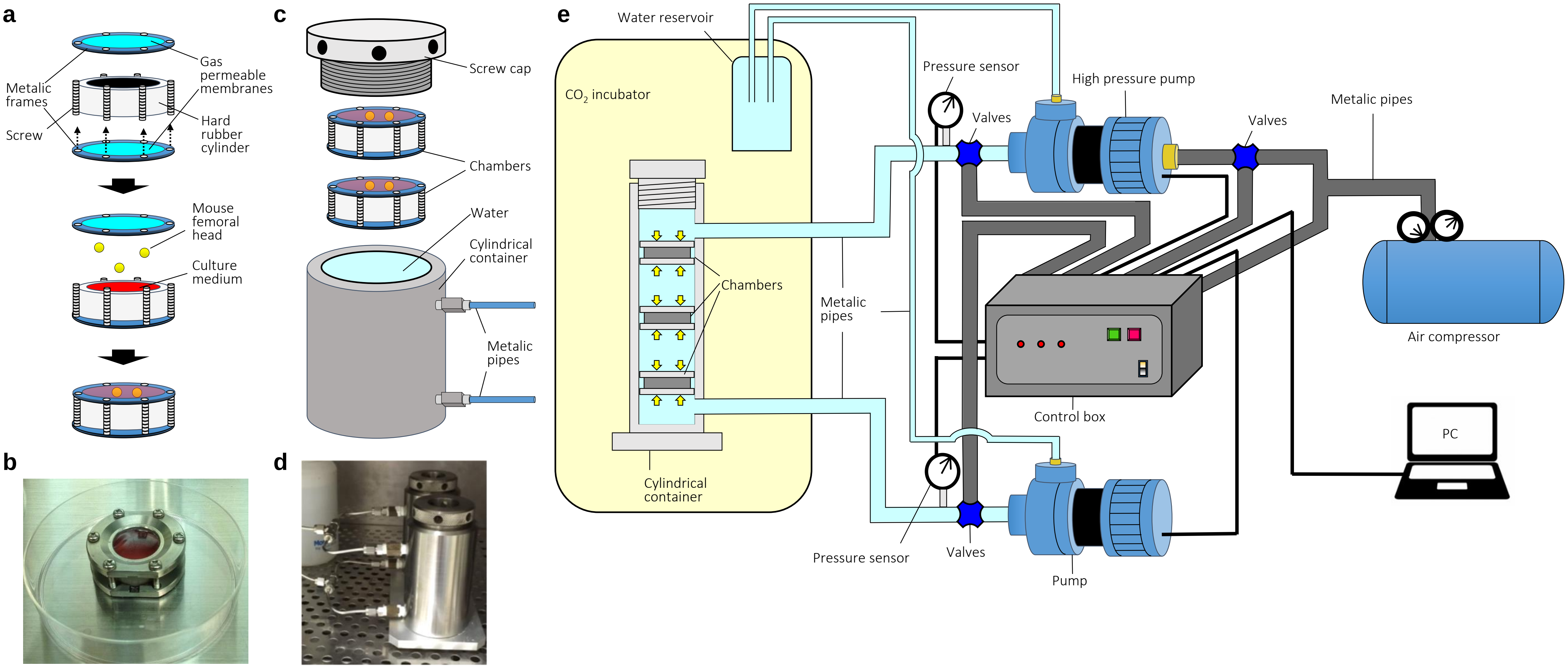

Supplementary Figure 1 | Schematic of the procedure for the cyclic hydrostatic pressure loading on mouse femoral head explants. (**a**) The procedure of placing mouse femoral heads into a chamber. After sterilized components were set up, culture medium and mouse femoral heads were put into the chamber and sealed in an aseptic condition. (**b**) Appearance of the sealed chamber on a 10-cm dish. (**c**) The procedure of placing the chambers containing culture medium and mouse femoral heads into a cylindrical container. The container is filled with water, and tightly sealed with a screw cap to prevent air bubble intrusion. The container can contain up to 5 chambers. (**d**) Appearance of the cylindrical containers in a CO_2 incubator. The present system can provide hydrostatic pressure to up to 2 containers. (**e**) A simplified circuit of the system. A high-pressure pump and the cylindrical containers are connected by metalic pipes filled with water, and regulated by several valves.

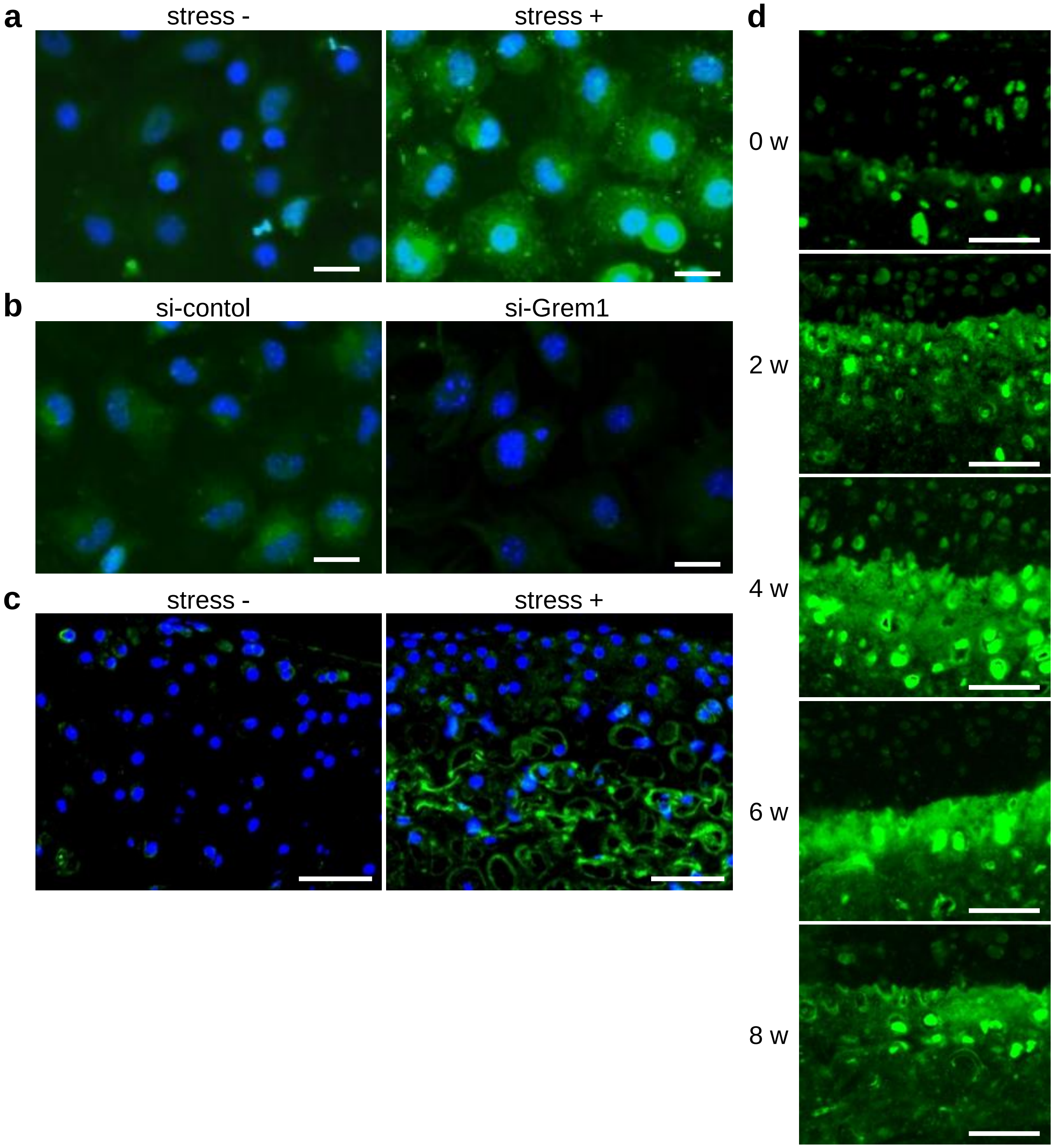

Supplementary Figure 2 | Induction of gremlin-1 protein confirmed by an antibody from a second manufacturer (R&D Systems). (**a**) Gremlin-1 protein expression in mouse primary chondrocytes 24 hours after tensile stress loading (stress +), or without loading (stress −). Nuclei were stained with DAPI (blue). Scale bars, 50 μm. (**b**) Gremlin-1 protein expression in mouse primary chondrocytes transfected with siRNA against gremlin-1 (si-Grem1) or control (si-control). Nuclei were stained with DAPI (blue). Scale bars, 50 μm. (**c**) Gremlin-1 immunofluorescence in mouse femoral head cartilage after 20 MPa hydrostatic pressure loading. Nuclei were stained with DAPI (blue). Scale bars, 100 μm. (**d**) Gremlin-1 immunofluorescence during the time-course of mouse osteoarthritis development after surgical induction. Scale bars, 50 μm.

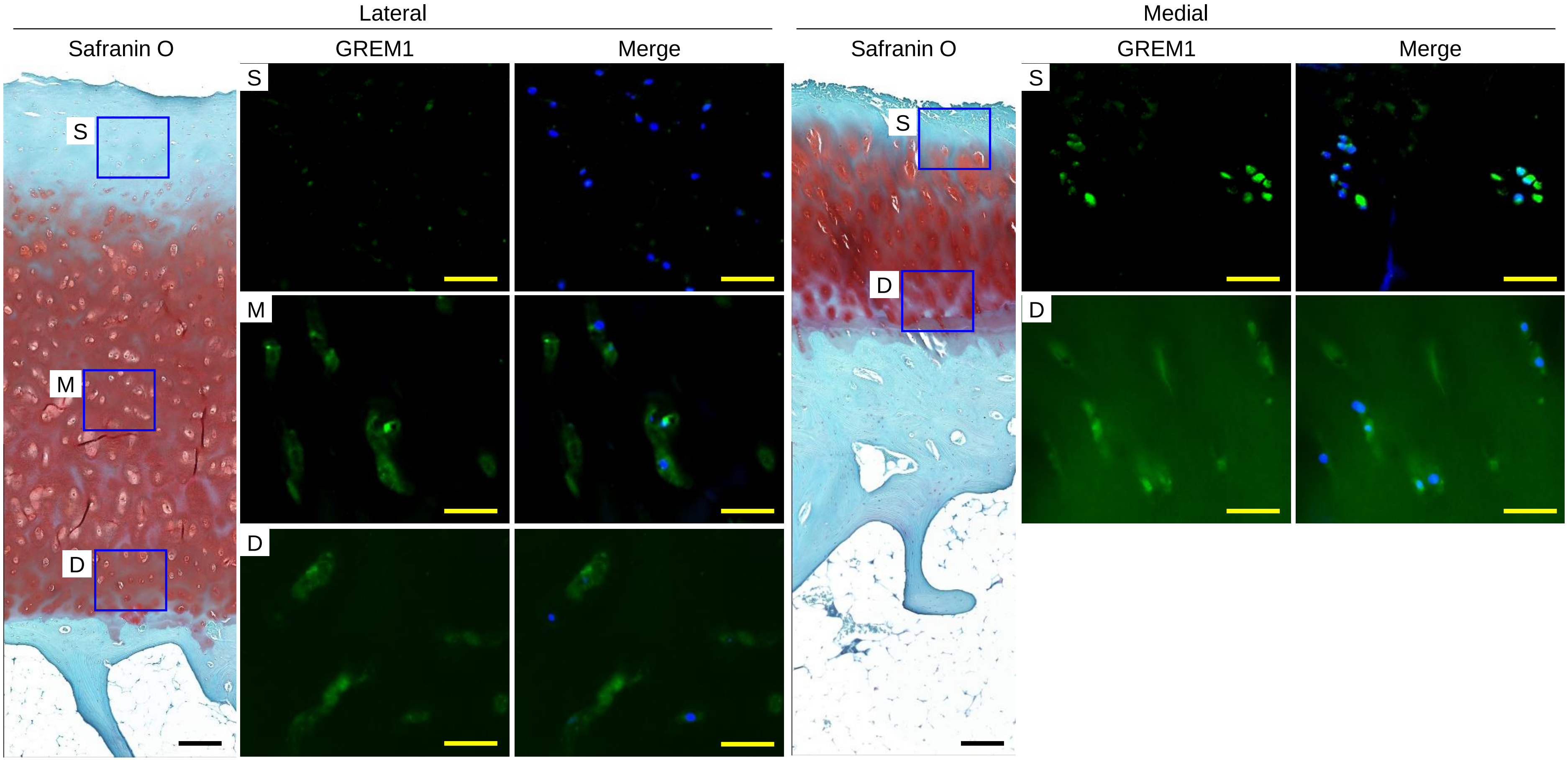

Supplementary Figure 3 | Gremlin-1 protein expression in human tibial articular cartilage surgical specimens from total knee arthroplasty. The lateral and medial compartments display mild and severe osteoarthritic changes, respectively. Inset boxes in safranin-O stainings indicate the region shown in the enlarged immunofluorescence of GREM1. S: superficial zone, M: middle zone, D: deep zone. Scale bars, 200 μm and 50 μm.

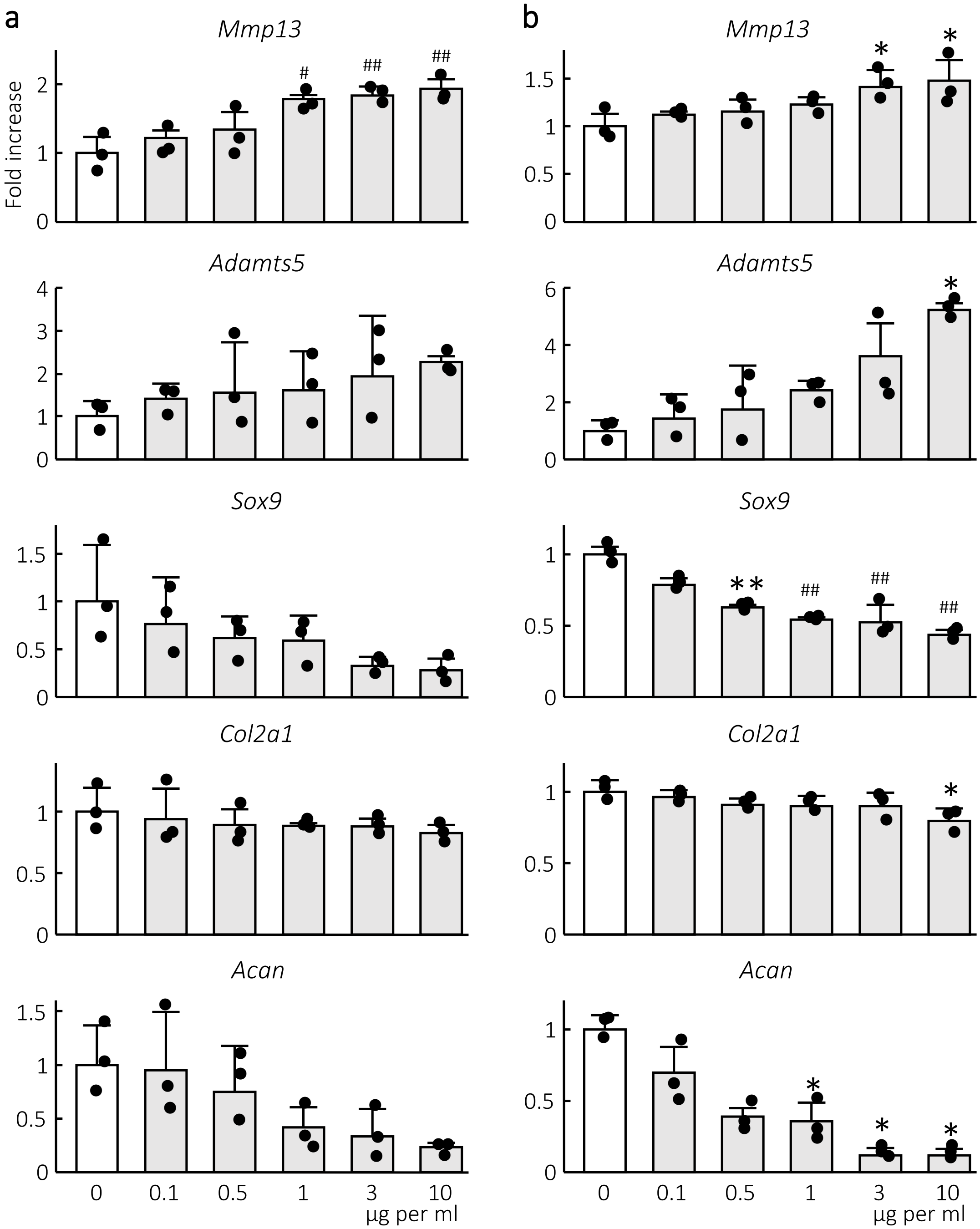

Supplementary Figure 4 | mRNA levels of marker genes in primary mouse articular chondrocytes treated with recombinant human gremlin-1 (PeproTech) for 4 hours (**a**) and 8 hours (**b**). All error bars are expressed as means ± SD of biologically independent three samples per group. *P < 0.05, **P < 0.01, #P < 0.005, ##P < 0.001 versus 0 (vehicle) (one-way ANOVA test) .

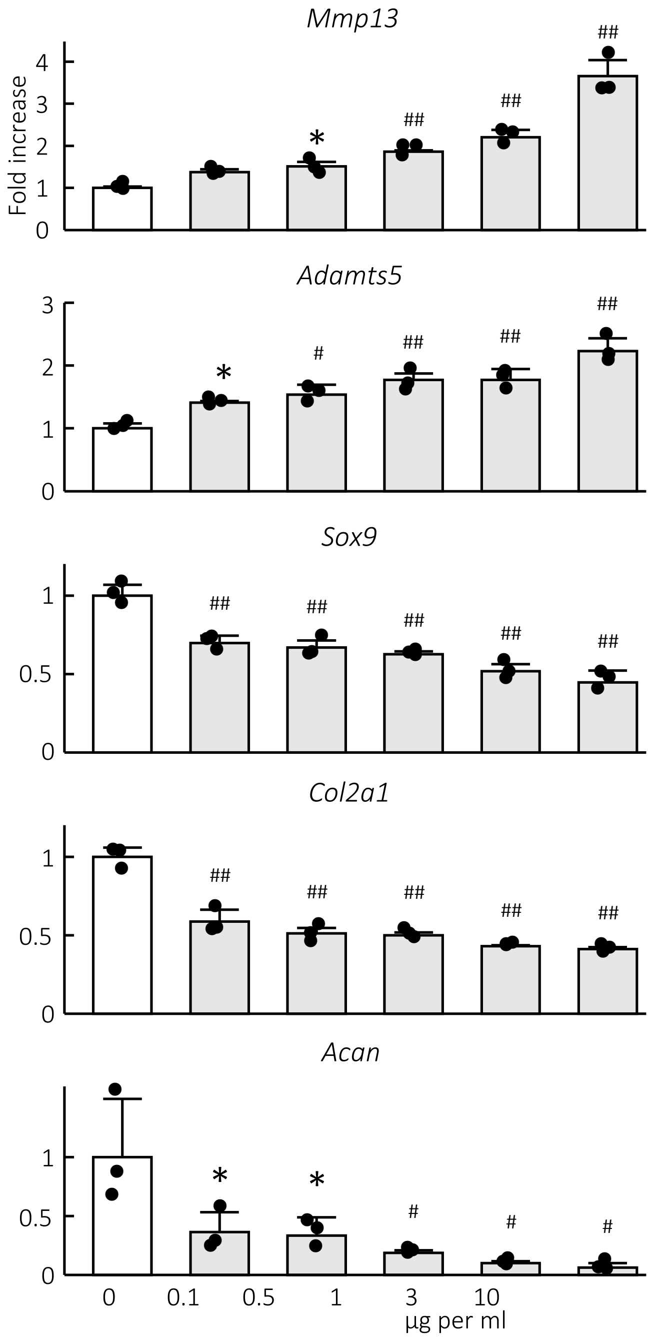

Supplementary Figure 5 | mRNA levels of marker genes in primary mouse articular chondrocytes treated with recombinant human gremlin-1 from a second manufacturer (R&D Systems) for 24 hours. All error bars are expressed as means ± SD of biologically independent three samples per group. **P* < 0.05, ***P* < 0.01, #*P* < 0.005, ##*P* < 0.001 versus 0 (vehicle) (one-way ANOVA test) .

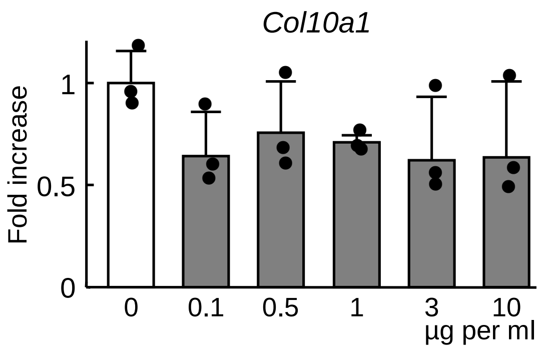

Supplementary Figure 6 | mRNA levels of Col10a1 in mouse primary chondrocytes treated with recombinant human gremlin-1 for 48 hours. All error bars are expressed as means ± SD of biologically independent three samples per group (one-way ANOVA test) .

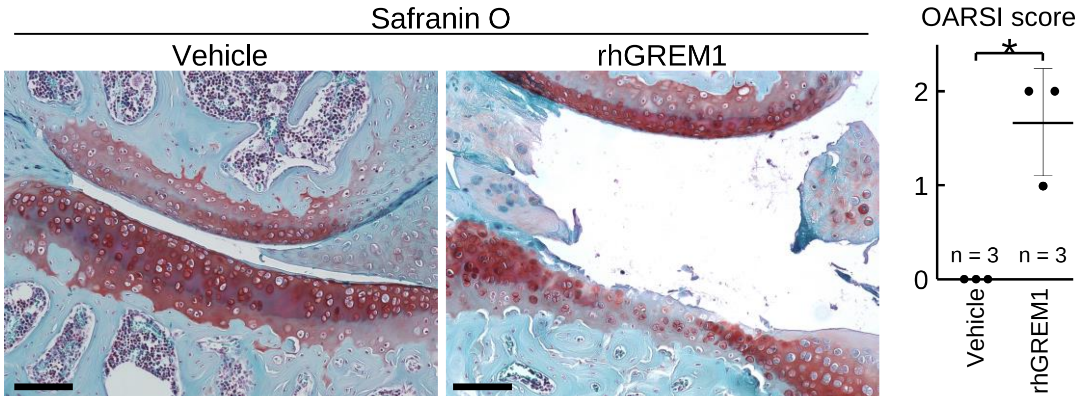

Supplementary Figure 7 | Effects of recombinant human gremlin-1 (rhGREM1) on normal mouse articular cartilage. Safranin O staining and OARSI scores of mouse knee joints after intra-articular administration (twice a week for 8 weeks) of 10 μL of 10 μg/mL rhGREM1 or vehicle. Scale bars, 100 μm. All error bars are expressed as means ± SD of biologically independent three animals per group. *$P < 0.05$ (Student’s unpaired two-tailed t-test).

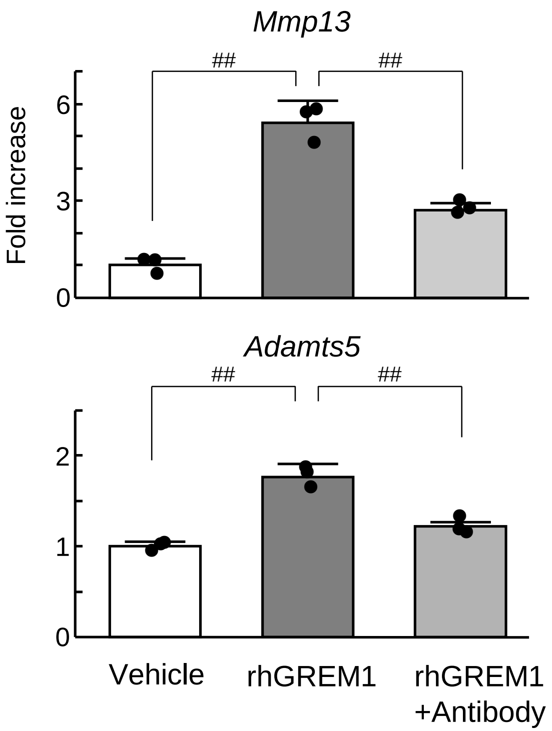

Supplementary Figure 8 | mRNA levels of Mmp13 and Adamts5 in mouse primary chondrocytes treated with recombinant human gremlin-1(rhGREM1) and antibody against Grem1. All error bars are expressed as means ± SD of biologically independent three samples per group. $^{\#\#}P < 0.001$ (one-way ANOVA test) .

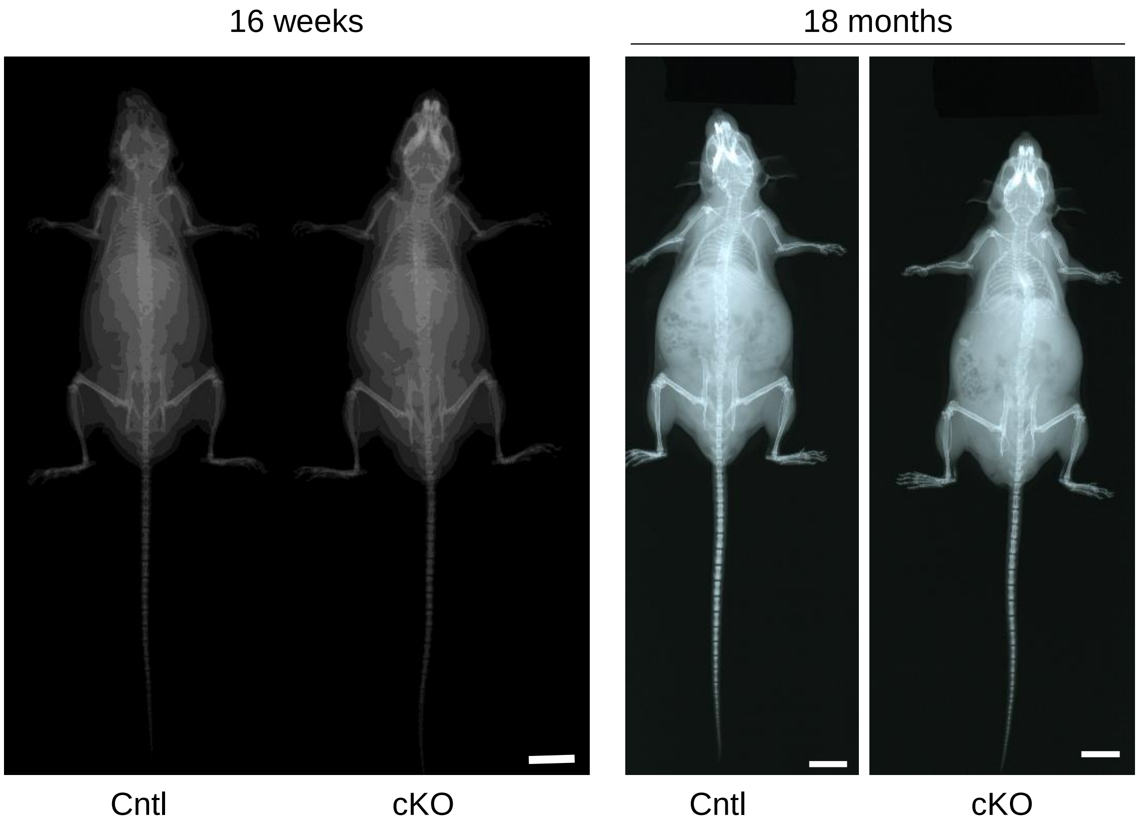

Supplementary Figure 9 | Plain radiograph of *Grem1*$^{fl/fl}$ (Cntl) and *Col2a1-Cre*^{ERT2}*;Grem1*$^{fl/fl}$ (cKO) mice at 16 weeks , and 18 months of age. Scale bars, 1 cm.

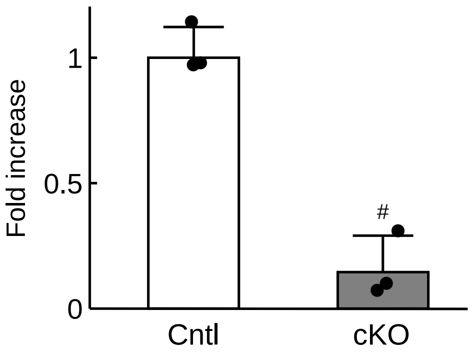

Supplementary Figure 10 | mRNA levels of gremlin-1 in knee joint articular cartilage of *Grem1*$^{fl/fl}$ (Cntl) and *Col2a1-Cre*^{ERT2}*;Grem1*$^{fl/fl}$ (cKO) mice at 16 weeks of age. Three mice were analysed for each group. Tamoxifen induction was performed at 7 weeks. All error bars are expressed as means ± SD of biologically independent three animals per group #$P < 0.005$ (Student's unpaired two-tailed t-test) .

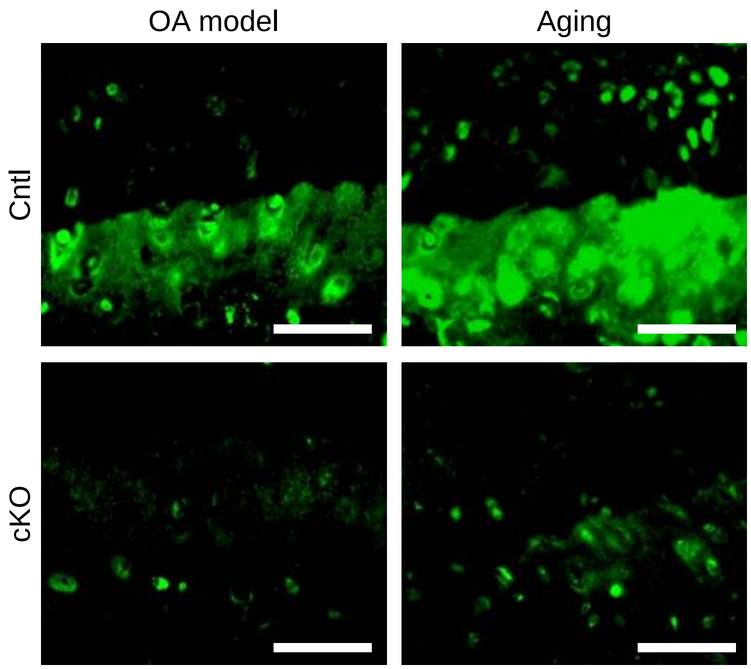

Supplementary Figure 11 | Expression of gremlin-1 protein in mouse knee joints of *Grem1*$^{fl/fl}$ (Cntl) and *Col2a1-Cre*^{ERT2}*;Grem1*$^{fl/fl}$ (cKO) mice 8 weeks after surgery (OA model), or at 18 months of age (Aging), confirmed by an antibody from a second manufacturer (R&D Systems). Tamoxifen induction was performed at 7 weeks. Scale bars, 50 µm.

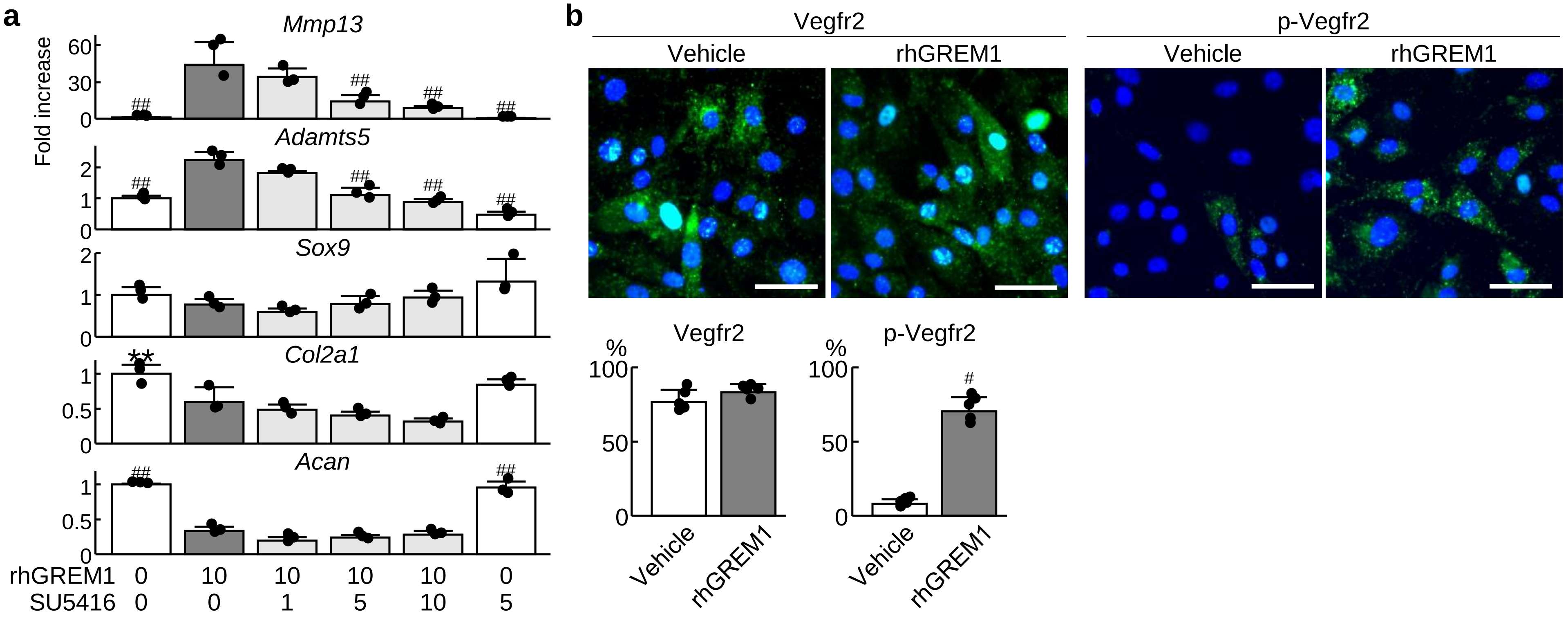

Supplementary Figure 12 | Involvement of VEGFR2 in catabolic effects of gremlin-1 (**a**) mRNA levels of marker genes in mouse primary chondrocytes treated with or without rhGREM1 (10 µg/mL) and VEGFR2 inhibitor SU5416 (1-10 µM). n=3 biologically independent samples. $^{**}P < 0.01$, $^{\#\#}P < 0.001$ versus rhGREM1 without SU5416 (one-way ANOVA test) . (**b**) Immunofluorescence and percentages of cells positive for VEGFR2 and p-VEGFR2 proteins in mouse primary chondrocytes cultured with or without 1 µg/mL rhGREM1 for 10 minutes. Nuclei were stained with DAPI (blue). Scale bars, 50 µm. N=5 biologically independent experiments. $^{\#}P < 0.005$ versus vehicle (Student’s unpaired two-tailed t-test) . All error bars are expressed as mean ± SD.

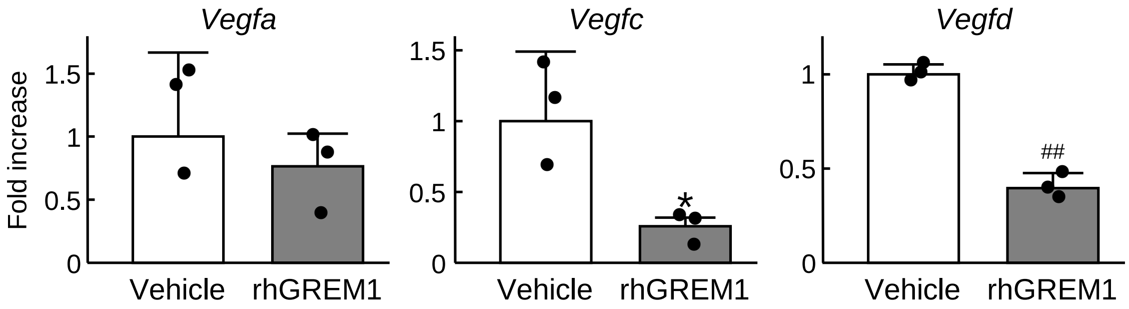

Supplementary Figure 13 | mRNA levels of VEGF family members in mouse primary chondrocytes treated with recombinant human gremlin-1 (rhGREM1) or vehicle for 48 hours. All error bars are expressed as means ± SD of biologically independent three samples per group. **P* < 0.05, $^{\#\#}P < 0.001$ (Student’s unpaired two-tailed t-test) .

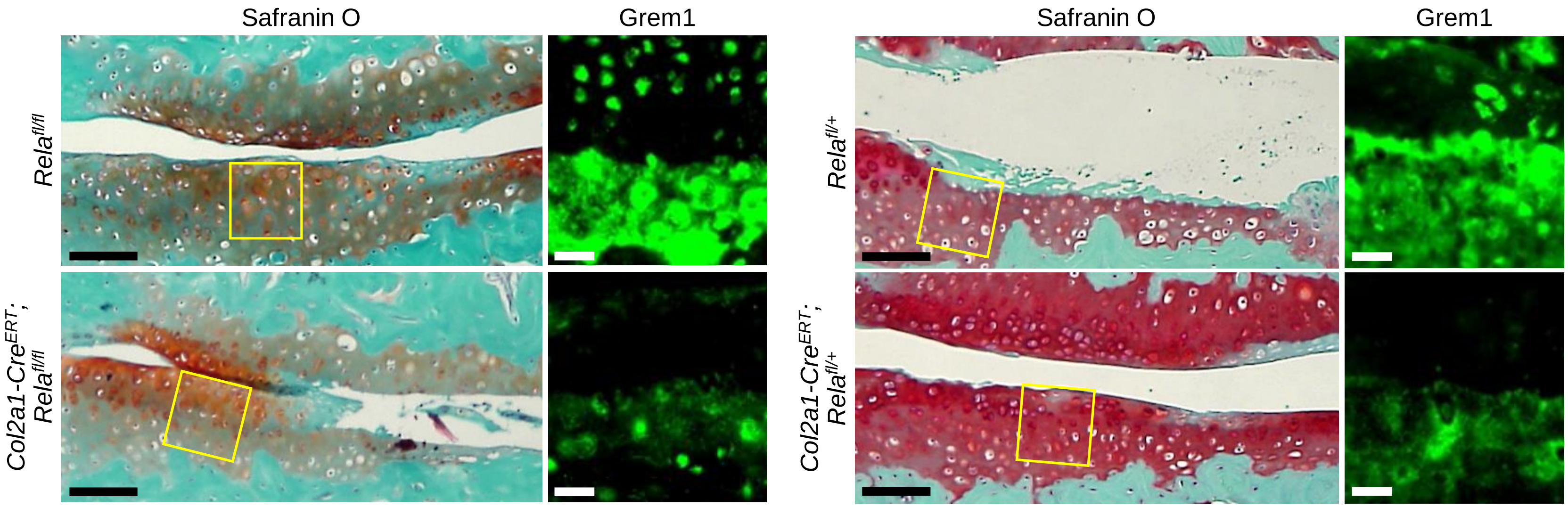

Supplementary Figure 14 | Grem1 expression in articular cartilage of *Rela*$^{fl/fl}$, *Col2a1-Cre*^{ERT}*;Rela*$^{fl/fl}$, *Rela*$^{fl/+}$, and *Col2a1-Cre*^{ERT}*;Rela*$^{fl/+}$ mice 8 weeks after OA induction. Inset boxes indicate the regions of immunofluorescence. Scale bars, 100 and 20 µm, respectively.

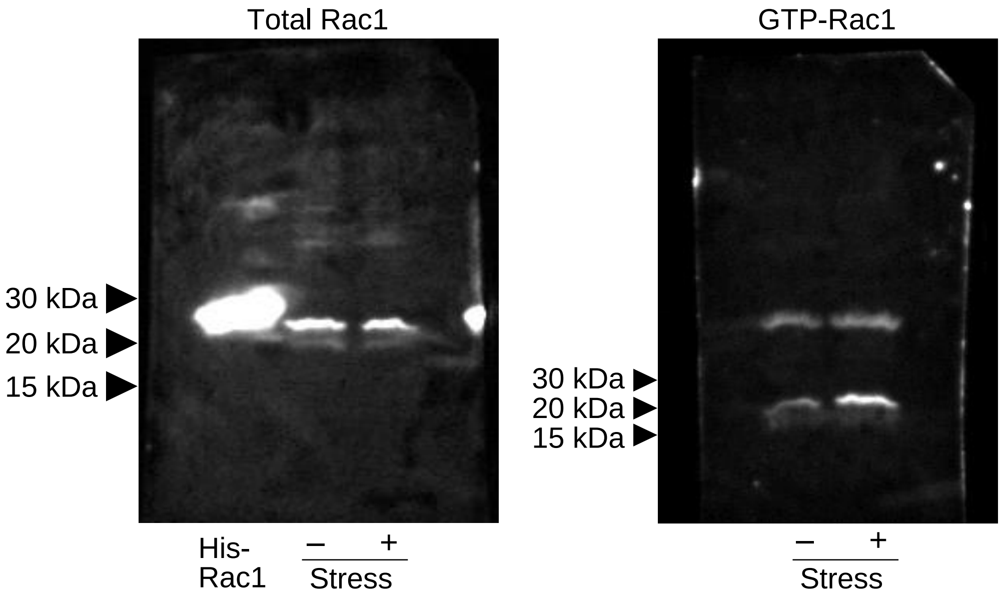

Supplementary Figure 15 | Original images of immunoblots shown in Fig. 7c. His-tagged Rac1 (His-Rac1) was used for control.

Gene Symbol	Accession	Signal intensity		Log2 Ratio
		before loading	after loading	
Capn11	NM_001013767	9299.055269	53325.7646	2.519676687
Msmp	NM_001099314	7407.455253	38810.71353	2.389405046
Krtap8-2	NM_010676	3469.265334	17042.34407	2.296421693
March9	NM_001033262	4571.987704	17082.54994	1.901629916
Grem1	NM_011824	5698.025738	16749.12464	1.555551655
Efemp1	NM_146015	6431.176527	15681.79843	1.285936426
Trpc2	NM_011644	4620.660478	10866.69348	1.233742033
Frzb	NM_011356	9014.812503	20010.49583	1.150387525
Tubb2b	NM_023716	17862.49242	39118.77646	1.130927848
Ogn	NM_008760	26155.23312	54398.34715	1.056463188
Lgals3bp	NM_011150	6845.96582	14122.8949	1.044709847
Dcaf12l1	NM_178739	4924.365956	10046.45043	1.028675979

Supplementary Table 1 | List of abundantly expressing genes upregulated more than twice by cyclic tensile strain loading. Genes with signal intensity after loading ≧10,000 were shown.

Gene Symbol	Accession	Signal intensity		Log2 Ratio
		before loading	after loading	
Cdca3	NM_013538	10502.7502	1932.064101	-2.44255229
Ccnb2	NM_007630	10629.26583	2010.882243	-2.402141447
Plk1	NM_011121	12939.87599	2829.856348	-2.193023067
Cdc20	NM_023223	16180.12155	3670.30849	-2.140249214
Birc5	NM_001012273	20131.94976	4742.967313	-2.085625072
Cdca8	NM_026560	10556.36363	2551.777252	-2.048538648
Col10a1	NM_009925	37809.16653	13367.92945	-1.499960022
S100a4	NM_011311	37928.23417	13598.34897	-1.479840707
Anxa8	NM_013473	44633.29228	16806.4497	-1.409105235
S100a4	NM_011311	61894.92197	23460.04822	-1.399615073
Spc25	NM_001199123	17662.13086	7151.440164	-1.304353701
Stmn1	NM_019641	37199.01232	15659.03743	-1.248268784
Slpi	NM_011414	11671.38066	4978.239164	-1.229267788
Mt2	NM_008630	289979.607	124420.5658	-1.220726474
Hist1h2bm	NM_178200	25352.11135	11443.95537	-1.147520124
D2Ertd750e	NM_026412	59395.60108	27927.09463	-1.088690595
Hist1h2ba	NM_175663	14721.8378	7012.534601	-1.069949892
Hmgb2	NM_008252	18277.99952	8720.495652	-1.067626138
Mfsd2b	NM_001033488	349494.0358	166924.5508	-1.066071678
Stmn1	NM_019641	12581.30742	6031.354698	-1.060727865
Hist1h2ag	NM_178186	117049.5754	56354.92292	-1.054506152
S100a6	NM_011313	224818.5125	109290.3577	-1.040594716
Hist1h2ab	NM_175660	19409.99793	9466.673736	-1.035870458
Incenp	NM_016692	10455.74697	5226.357105	-1.000418525

Supplementary Table 2 | List of abundantly expressing genes downregulated more than twice by cyclic tensile strain loading. Genes with signal intensity before loading ≧10,000 were shown.

Gene Symbol	Accession	Signal intensity		Log2 Ratio
		before loading	after loading	
Grem1	NM_011824	5698.025738	16749.12464	1.555551655
Clu	NM_013492	19884.8569	36828.53628	0.889153879
Tspan6	NM_019656	43056.00086	60572.69499	0.492453276
Gstp1	NM_013541	7994.933624	11047.99402	0.466626483
S100b	NM_009115	108924.823	148680.8974	0.448886531
Gstp1	NM_013541	13734.66203	18432.17269	0.424404728
Slc44a2	NM_152808	24591.25809	32459.21663	0.400482637
Hspb1	NM_013560	10850.31176	14208.69491	0.38903755
Prdx2	NM_011563	31153.23507	40689.33052	0.385268557
Shisa5	NM_025858	35668.36822	45684.58787	0.357062324
Ptplad1	NM_021345	21919.26412	27062.23038	0.304081382
Traf3	NM_011632	9490.775175	11461.2271	0.272163683
Flna	NM_010227	55299.53406	66460.08773	0.265220873
Mib2	NM_001256107	13798.96856	16480.43276	0.256193694
Fbxw11	NM_134015	23505.87301	27996.04548	0.252201794
Prmt2	NM_001077638	10457.81841	12396.85821	0.245392614
Tlr9	NM_031178	11366.54549	13433.3369	0.241023863
Dab2ip	NM_001114125	10347.36042	12187.82273	0.236177633
Pink1	NM_026880	9232.91924	10848.95177	0.232696884
Fkbp1a	NM_008019	156737.4314	183032.5624	0.223750574

Supplementary Table 3 | List of top 20 NF-kB-related genes upregulated by cyclic tensile strain loading. Genes with signal intensity after loading ≧10,000 were shown.

Gene Symbol	Accession	Signal intensity		Log2 Ratio
		before loading	after loading	
S100a4	NM_011311	37928.23417	13598.34897	-1.479840707
Hmox1	NM_010442	37904.40065	24122.36805	-0.651993812
Nup62	NM_053074	24734.81908	16256.28992	-0.60554531
Ecm1	NM_007899	19558.0677	13999.76867	-0.482360853
Npm1	NM_008722	166486.5224	128292.0788	-0.375973295
Ndfip1	NM_022996	15024.6955	11901.28195	-0.336218771
Siva1	NM_013929	16156.08808	13032.04294	-0.310014654
Lgals1	NM_008495	76556.75807	61962.53392	-0.305133594
Slc20a1	NM_015747	30362.68988	24778.31843	-0.293221324
Commd7	NM_001195390	14246.93684	12219.55867	-0.221459585
Prdx1	NM_011034	48416.10442	42244.75456	-0.196714788
Commd1	NM_144514	12011.47625	10806.53824	-0.152509029
Litaf	NM_019980	12327.85029	11162.25455	-0.143292794
Traf2	NM_009422	14829.6365	13587.03237	-0.126252853
Rps3	NM_012052	173236.8482	159272.511	-0.121248538
Psma6	NM_011968	21219.09437	19777.72847	-0.101486345
Irak2	NM_172161	14387.37068	13882.18531	-0.051568268
Rhoa	NM_016802	39887.70175	38727.06553	-0.042601812
Arhgef2	NM_001198911	32715.71052	32091.54059	-0.027790554
Ndfip1	BC020359	47294.20926	46961.15776	-0.010195573

Supplementary Table 4 | List of top 20 NF-kB-related genes downregulated by cyclic tensile strain loading. Genes with signal intensity before loading ≧10,000 were shown.

Gene Symbol	Species		Sequence
Gapdh	mouse	F	tgcaccaccaactgcttagc
		R	ggatgcagggatgatgttct
Mmp13	mouse	F	aggccttcagaaaagccttc
		R	tccttggagtgatccagacc
Grem1	mouse	F	tggagaggaggtgcttgagt
		R	aacttcttgggcttgcagaa
Adamts5	mouse	F	gctactgcacagggaagagg
		R	tgcatgtttgggaacccatt
Sox9	mouse	F	cgactacgctgaccatcaga
		R	agactggttgttcccagtgc
Col2a1	mouse	F	gccaagacctgaactctgc
		R	gccatagctgaagtggaagc
Acan	mouse	F	ccaaaccagcctgacaactt
		R	tctagcatgctccaccactg
Vegfa	mouse	F	caggctgctgtaacgatgaa
		R	tttcttgcgctttcgttttt
Vegfc	mouse	F	tttcctgtgaggctcgtacc
		R	acgccagagacaagaagcac
Vegfd	mouse	F	gtatggactcacgctcagca
		R	ctccacaccggaagacattt
RAC1	human	F	aaccaatgcatttcctggag
		R	tgtttgcggataggataggg

Supplementary Table 5 | Primers used for qRT-PCR.
