# Supplementary material for: Excessive mechanical loading promotes osteoarthritis through the gremlin-1–NF-κB pathway
Source: Nat Commun. 2019 Mar 29;10:1442. doi: 10.1038/s41467-019-09491-5 (PMC6441020; doi:10.1038/s41467-019-09491-5)
Supplement: Supplementary file 2 — Reporting Summary [file 41467_2019_9491_MOESM2_ESM.pdf]

nature research

Corresponding author(s): Taku Saito

Last updated by author(s): Feb 7, 2019

Reporting Summary

Nature Research wishes to improve the reproducibility of the work that we publish. This form provides structure for consistency and transparency in reporting. For further information on Nature Research policies, see Authors & Referees and the Editorial Policy Checklist.

Statistics

For all statistical analyses, confirm that the following items are present in the figure legend, table legend, main text, or Methods section.

n/a	Confirmed	
☐	☒	The exact sample size (*n*) for each experimental group/condition, given as a discrete number and unit of measurement
☐	☒	A statement on whether measurements were taken from distinct samples or whether the same sample was measured repeatedly
☐	☒	The statistical test(s) used AND whether they are one- or two-sided<br>*Only common tests should be described solely by name; describe more complex techniques in the Methods section.*
☒	☐	A description of all covariates tested
☒	☐	A description of any assumptions or corrections, such as tests of normality and adjustment for multiple comparisons
☐	☒	A full description of the statistical parameters including central tendency (e.g. means) or other basic estimates (e.g. regression coefficient) AND variation (e.g. standard deviation) or associated estimates of uncertainty (e.g. confidence intervals)
☒	☐	For null hypothesis testing, the test statistic (e.g. *F*, *t*, *r*) with confidence intervals, effect sizes, degrees of freedom and *P* value noted<br>*Give P values as exact values whenever suitable.*
☒	☐	For Bayesian analysis, information on the choice of priors and Markov chain Monte Carlo settings
☒	☐	For hierarchical and complex designs, identification of the appropriate level for tests and full reporting of outcomes
☒	☐	Estimates of effect sizes (e.g. Cohen's *d*, Pearson's *r*), indicating how they were calculated

Our web collection on statistics for biologists contains articles on many of the points above.

Software and code

Policy information about availability of computer code

Data collection	none
Data analysis	JMP Pro 13 was used for data analysis.

For manuscripts utilizing custom algorithms or software that are central to the research but not yet described in published literature, software must be made available to editors/reviewers. We strongly encourage code deposition in a community repository (e.g. GitHub). See the Nature Research guidelines for submitting code & software for further information.

Data

Policy information about availability of data

All manuscripts must include a data availability statement. This statement should provide the following information, where applicable:

- Accession codes, unique identifiers, or web links for publicly available datasets
- A list of figures that have associated raw data
- A description of any restrictions on data availability

The raw microarray data are deposited in the Gene Expression Omnibus (www.ncbi.nlm.nih.gov/geo/) under accession no. GSE103159. All other data supporting the findings of this study are available within the article and the Supplementary Information file, or are available from the corresponding author on reasonable request.

Field-specific reporting

Please select the one below that is the best fit for your research. If you are not sure, read the appropriate sections before making your selection.

☒ Life sciences ☐ Behavioural & social sciences ☐ Ecological, evolutionary & environmental sciences

For a reference copy of the document with all sections, see nature.com/documents/nr-reporting-summary-flat.pdf

Life sciences study design

All studies must disclose on these points even when the disclosure is negative.

Sample size	No statistical methods were used to predetermine sample sizes. All sample numbers were stated in figure legends.
Data exclusions	No data was excluded, unless reasonably shown to be contaminated, degraded (either sample or reagents)
Replication	All experiments were replicated in at least 3 independent experiments.
Randomization	The experiments were not randomized
Blinding	Image analysis (scoring of OARSI score of mice) was done blind.

Reporting for specific materials, systems and methods

We require information from authors about some types of materials, experimental systems and methods used in many studies. Here, indicate whether each material, system or method listed is relevant to your study. If you are not sure if a list item applies to your research, read the appropriate section before selecting a response.

Materials & experimental systems

n/a	Involved in the study	
☐	☒	Antibodies
☐	☒	Eukaryotic cell lines
☒	☐	Palaeontology
☐	☒	Animals and other organisms
☒	☐	Human research participants
☒	☐	Clinical data

Methods

n/a	Involved in the study	
☒	☐	ChIP-seq
☒	☐	Flow cytometry
☒	☐	MRI-based neuroimaging

Antibodies

Antibodies used	gremlin-1(LS-C125371, LSbio), gremlin-1(AF956, R&D Systems), Mmp13 (MAB13426, Chemicon), Adamts5 (H-200, Santa Cruz Biotechnology), IκBα (sc-371, Santa Cruz Biotechnology), p-IκBαS32/36 (sc-101713, Santa Cruz Biotechnology), HIF-2α (NB100-122, Novus Biologicals),VEGFR2 (55B11, Cell Signaling Technology), p-VEGFR2 (Tyr951, #2471, Cell Signaling Technology), AlexaFluor 488-conjugated mouse and rabbit secondary antibodies (nvitrogen),Rac1 (ARC03,Cytoskeleton)
Validation	Validation statements are noted on the manufacturer website

Eukaryotic cell lines

Policy information about cell lines

Cell line source(s)	ATDC5 cells (RIKEN Cell Bank)
Authentication	The identities of the cell lines were not authenticated. The mutations in the cell lines were validated by next generation and Sanger sequencing.
Mycoplasma contamination	No test for mycoplasma contamination was performed.
Commonly misidentified lines (See ICLAC register)	None of these cell lines were utilized.

Animals and other organisms

Policy information about studies involving animals; ARRIVE guidelines recommended for reporting animal research

Laboratory animals	C57BL/6J mice aged 8 to 12 weeks and 18 months both male and female, were used in the study.
Wild animals	not applicable
Field-collected samples	not applicable
Ethics oversight	All experiments were performed according to protocols approved by the Animal Care and Use Committee of The University of Tokyo. We have complied with all relevant ethical regulations.

Note that full information on the approval of the study protocol must also be provided in the manuscript.
